# Gut microbiota associated with equol production in school-age children

**DOI:** 10.1007/s00394-025-03625-w

**Published:** 2025-05-09

**Authors:** Keiko Wada, Wataru Suda, Tomomi Ueno, Hiroaki Masuoka, Michiyo Yamakawa, Yuma Nakashima, Masaaki Sugino, Tomoka Mori, Shigeto Uchiyama, Yoshio Sumoto, Yuya Kiguchi, Masahira Hattori, Chisato Nagata

**Affiliations:** 1 Department of Epidemiology and Preventive Medicine, Gifu University Graduate School of Medicine, 1-1 Yanagido, Gifu City, Gifu 501-1194 Japan; 2 Laboratory for Microbiome Sciences, RIKEN Center for Integrative Medical Sciences, Yokohama, Japan; 3 Saga Nutraceuticals Research Institute, Otsuka Pharmaceutical Co., Ltd., Saga, Japan; 4 Department of Social Studies Education, Graduate School of Education, Gifu University, Gifu, Japan; 5 Department of Computational Biology and Medical Sciences, Graduate School of Frontier Sciences, The University of Tokyo, Tokyo, Japan; 6 School of Advanced Science and Engineering, Waseda University, Tokyo, Japan

**Keywords:** Gut microbiota, Isoflavone, Equol, Food frequency questionnaire, Children, Epidemiology

## Abstract

**Purpose:**

Little is known about the relationship between the human gut microbiota composition and equol-producing ability. The aim of this study is to evaluate the relationship between equol production and the gut microbiota in school-age children, with consideration of sex, age, and exposure to soy isoflavones.

**Methods:**

Participants were 1110 students aged 7–8, 10–11 and 13–14 years. The cumulative participation rate was 85.2%. Equol production, defined as a log-transformed equol/daidzein ratio of −1.75 or greater, was determined in urine collected after two days of soymilk supplementation. Urinary daidzein, genistein, and equol were measured by liquid chromatography–mass spectrometry. The microbiota in 223 fecal samples was determined by 16S rRNA gene sequencing analysis.

**Results:**

The observed operational taxonomic unit number, Chao 1, and Shannon index were significantly higher in equol producers than in non-producers after adjustments for survey time, sex, and age. Principal coordinate analysis plots based on weighted and unweighted UniFrac distances showed significant differences in the gut microbiota between equol producers and non-producers. Among the species with an average abundance of ≥0.1%, several species were more abundant in equol producers than in non-producers, with significant positive correlations between relative abundances and equol/daidzein ratios. Of species previously reported as equol-producing, *Asaccharobacter celatus* and *Slackia isoflavoniconvertens* were significantly associated with equol production.

**Conclusion:**

Equol production was strongly associated with the diversity of the gut microbiota. The richness of a diverse microbiota and the interrelationships among microbes may be involved in equol production.

**Supplementary Information:**

The online version contains supplementary material available at 10.1007/s00394-025-03625-w.

## Introduction

Consumption of soy foods is associated with decreased risks of heart disease, diabetes, and several cancers [1–6]. Isoflavones, major phytochemicals in soybeans, are reported to have antioxidative, anti-inflammatory, and anti-cancer properties in animal models and *in vitro* studies [7–9]. Because isoflavones are structurally similar to estrogens, they may also affect human hormone metabolism by binding to estrogen receptors [10]. Equol has been demonstrated to have a much greater affinity for estrogen receptors and a higher antioxidant activity than other isoflavone metabolites [11, 12], implicating in more health benefits than the others.

In humans, equol is produced from daidzein by metabolic actions of the gut microbiome [13]. However, equol-producing ability varies widely among individuals, which may be largely related to the host genetics and/or the gut microbiome. The percentage of equol producers depends on the population and their diet, and in Japan it is reported to be around 20–40 % in adults [14–16]. Microbial colonization begins immediately after birth in the gastrointestinal tract [17]. The infant gut microbiome is highly variable compared with the relatively stable gut microbiome of adults [18]. Equol is not detected in the urine of most infants at six months of age [19], and the positive rate of equol in the urine is lower in children than in adults [14]. In addition, similar to equol, phytoestrogen metabolites in feces are incomplete in children compared with adults [20]. From these findings, equol-producing ability is thought to be developed during childhood when the gut microbiome is shifted to the adult type. Therefore, it is worth understanding the relationship between equol production and the gut microbiome in children. Although considerable efforts have been made to detect specific gut bacteria capable of producing equol [13, 21], little is known about the association between them.

Thus, the aim of this study is to investigate the relationship between equol production and gut microbiota in school-age Japanese children. The relationship was evaluated with consideration of sex, age, and exposure to soy isoflavones.

## Methods

### Participants and design

This cross-sectional study was conducted annually from 2013 to 2016 at a public elementary and a junior high school in Gifu, Japan. Subjects were elementary school students aged 7–8 and 10–11 years (the 2nd and 5th grade), respectively, and junior high school students aged 13–14 years (the 8th grade). Of the 427, 477, and 659 students in the 2nd, 5th, and 8th grades, respectively, 377, 402, and 553 students participated in the study (Supplementary Figure 1). The overall participation rate was 85.2%. Written informed consent was obtained from the participants’ parents or guardians before the study. The parents or guardians were asked to complete questionnaires, including the food frequency questionnaire (FFQ). During approximately the same periods, the participants provided urine and fecal samples. The study protocols and procedures involving the participants were approved by the ethics board of the Gifu University Graduate School of Medicine, Gifu, Japan (approval number: 30–084).

The 2nd and 5th grade students in the 2013 survey participated again in the 2016 survey in the 5th and 8th grade, respectively. To avoid using the same individuals twice in the analysis for the whole three age groups, the data from the 2nd and 5th grade students in the 2013 survey were excluded, as the 2016 survey included fecal sample collection. Finally, 1110 were included in the analysis, for which dietary isoflavone and urinary isoflavone levels were measured (Supplementary Figure 1).

### Estimation of isoflavone intake

Dietary intake was assessed using a validated FFQ [22, 23]. In the FFQ, the parents were asked how often their children consumed each of the food items listed and what the usual portion size of each item was during the past 6 months. An item on the average amount of food the children consumed at school lunches was also included. The food list contained 162 items for the 2nd-grade children and 163 items (plus coffee) for the 5th- and 8th-grade children. Food intake was estimated from the frequency of ingestion and the portion size, using the Japanese Standard Table of Food Composition (5th revised and enlarged edition) published by the Science and Technology Agency of Japan [24]. The isoflavone intake from soy products was estimated from their isoflavone concentration [25]. When compared with two 3-day diet records, the Spearman correlation coefficients for total energy and soy products were 0.34 and 0.30 in the FFQ for the 2nd-grade children, and 0.28 and 0.25 in the FFQ for the 5th- and 8th-grade children, respectively [22, 23]. The intakes of soy products and isoflavones were controlled for total energy by using the residual method proposed by Willett [26].

### Measurement of urinary equol excretion

Urine collection was performed twice. First, the participants were asked to provide first-morning-void urine once during the 3–5 day collection period (the first collection). About one week later, they were given a pack (125 mL) of soymilk as part of their school lunch on two consecutive days. The commercially available soymilk (MAMEPIYO^®^; MARUSAN-AI Co., Ltd., Aichi, Japan) contained 28–30 mg isoflavones. On the morning after the 2-day soy supplementation, another urine sample was collected (the second collection). Plastic containers of 20 mL (10 mL ×2) and 10 mL were used for the first and second urine samples, respectively. The urine samples were brought to school in the morning, immediately transported to our laboratory, and stored frozen at −80°C until the measurements of isoflavones and metabolites in 2021. Urinary isoflavones are reported to be stable at room temperature for 14 days [27].

Daidzein, genistein, glycitein and equol levels in urine were measured by liquid chromatography–mass spectrometry (LC–MS). Other precursors, intermediates, or metabolites of isoflavones were not measured. The details of the urine sample preparation and analysis procedures are described in Supplementary Material 1. The quantification limit for urine analysis was 2.5 ng/mL for equol and glycitein, and 10.0 ng/mL for daidzein and genistein. The inter-assay coefficients of variation determined from analysis of quality-control urine samples were 6.5% for equol and daidzein, 6.7% for genistein, and 5.9% for glycitein. Urinary creatinine was measured using the conventional enzymatic method at SRL, Inc. (Tokyo, Japan). To adjust for variation in the dilution of urine, urinary isoflavone levels were corrected for urinary creatinine levels.

The equol production positive (equol producers) were defined to those with a log-transformed equol/daidzein ratio of ≥ −1.75 [28]. One sample that showed less than the quantification limit of urinary daidzein was judged as equol production positive because the equol/daidzein ratio was greater than −1.75 when the quantification limit (10.0 ng/mL) was substituted for daidzein. When urinary equol was less than the quantification limit (190 samples), the equol/daidzein ratio was calculated by substituting the quantification limit (2.5 ng/mL), and when the ratio was less than −1.75, it was judged as equol production negative (184 samples). When the ratio was −1.75 or greater, it was judged as undeterminable (6 samples).

For implementation reasons, soymilk supplementation was not performed for the 2nd-grade students in 2016. Thus, 1106 and 972 urine samples were obtained at the first and the second collections, respectively, and equol production was determined for 1100 and 972 urine samples (Supplementary Figure 1).

### Analysis of fecal microbiota

In the 2016 survey, participants were asked to collect about 0.5 g of fecal samples at home. The fresh feces were immediately put in a container with stabilization solution by guanidine thiocyanate (MORA extract^®^; AMR Inc., Gifu, Japan). The container with feces was placed in envelopes, posted the same day, and transported to our laboratory within approximately one day. Immediately after arrival at the lab, the feces were stored at −80 ℃ until analysis.

The gut microbiota was analyzed by sequencing the 16S rRNA gene V1-V2 region amplified with 27Fmod and 338R primers [29]. The details of the fecal DNA preparation and analysis procedures are described in Supplementary Material 2. Among the filter-passed reads obtained, 10,000 high-quality reads per sample were randomly chosen for analysis. Total reads (the number of samples ×10,000) were grouped into operational taxonomic units (OTUs) using UCLUST (http://www.drive5.com/) with a sequence identity threshold of 97%. The representative sequences of the generated OTUs were subjected to a homology search against the database constructed from NCBI RefSeq 16S sequences (8/1/2020), using the GLSEARCH program for taxonomic assignments. For taxonomic assignment of OTUs at the phylum, genus, and species levels, sequence similarity thresholds of 70%, 94%, and 97% were applied, respectively. Alpha- and beta-diversity analyses were conducted using scikit-bio. For the alpha-diversity analysis, the observed OTU number, Chao1, and Shannon index were calculated. The UniFrac distance was used for beta-diversity analysis.

Of the 312 participants in the 2016 survey, 231 (74.0%) provided fecal samples (Supplementary Figure 1). After excluding participants who had taken antibiotics within 3 days prior to fecal collection, the gut microbiota was analyzed for 223 participants. Their equol production was determined for 222 and 133 in the first and second urine samples, respectively, as the second urine sample was not provided by the 2nd-grade students in 2016.

### Statistical analysis

As equol production in the urine after soymilk supplementation is a more accurate indicator of the equol production phenotype, the results of the second urine samples were adopted to assess the relationship between equol production and gut microbiota. However, the results of the first urine samples were presented as supplementary information for three reasons: the fecal samples were collected closer in time to the first urine samples than to the second urine samples, and the number of the first samples was greater than that of the second samples, and, in this Japanese population that consumes soy products daily, the equol production phenotypes in the first and second samples were similar.

First, sex and age were compared between equol producers and non-producers using *χ*^*2*^ test. The associations of soy isoflavone intakes and urinary isoflavone levels with equol production were evaluated by analysis of covariance (ANCOVA) with adjustment for survey time (survey year (2013, 2014, 2015, 2016) and grade (2nd, 5th, 8th)), sex (male, female), and age (years, continuous). Second, the associations of sex and age with microbiome alpha-diversity (observed OTU number, Chao1, and Shannon index) were evaluated by *t*-test or analysis of variance. The three alpha-diversity indices were compared by equol production and tertiles of isoflavone intake using ANCOVA with adjustment for survey time, sex, and age. Principal coordinate analysis (PCoA) with weighted and unweighted UniFrac distances was performed to visualize the dissimilarities in overall microbiota composition, and a permutational multivariate analysis of variance (PERMANOVA) was used to test for the differences in beta-diversity. The gut microbiota with differences in relative abundance between equol producers and non-producers were examined using the Wilcoxon rank sum test. The phylum and genus with an average abundance of ≥0.2% and the species with an average abundance of ≥0.1% of the participants were explored to focus on major components of overall microbiota and reduce the total number of statistical tests. *P*-values were adjusted for multiple testing using the Benjamini–Hochberg procedure.

We additionally examined bacterial species previously reported in the literature as equol-producing species [13, 21]. Twelve species were identified in our fecal samples: *Asaccharobacter celatus, Bacteroides ovatus, Bifidobacterium breve, Bifidobacterium longum, Finegoldia magna, Limosilactobacillus mucosae, Lacticaseibacillus paracasei, Latilactobacillus sakei, Lactococcus garvieae, Proteus mirabilis, Slackia isoflavoniconvertens, and Streptococcus intermedius*. The relative abundances of these species were compared between equol producers and non-producers using the Wilcoxon rank sum test.

To further investigate which species are associated with equol productivity, Spearman’s correlation coefficients between the equol/daidzein ratios and the relative abundances of species were calculated.

SAS version 9.4 (SAS Institute, Cary, NC) and R version 4.1.0 (R Foundation for Statistical Computing, Vienna, Austria) were used for data analysis. All *p*-values were calculated using a two-sided test. A *p*-value of less than 0.05 was considered statistically significant.

## Results

The participants’ characteristics, including soy isoflavone intakes and urinary levels of isoflavones in the first and second urine samples, are presented as means (standard deviations) or numbers (%) (Table 1). The estimated daily intake of isoflavones was 33.1 mg. The dietary sources of isoflavones are shown in Supplementary Table 1. Of the first and second urine samples, 26.2% and 22.3% were equol production-positive, respectively. Of 962 participants whose equol levels were measured in both first and second urine samples, 891 (92.6%) had reproducible results for the equol production in both urine samples.

**Table 1:** Characteristics of study participants

	Total participants (n=1110)	Participants examined for gut biome (n=223)
Age,* n* (%)		
7–8 years old	268 (24.1%)	76 (34.1%)
10–11 years old	292 (26.3%)	77 (34.5%)
13–14 years old	550 (49.5%)	70 (31.4%)
Sex, *n* (%)		
Male	558 (50.3%)	107 (48.0%)
Female	552 (49.7%)	116 (52.0%)
Height, mean (s.d.), cm	146.5 (15.3)	141.5 (14.4)
Weight, mean (s.d.), kg	39.5 (12.5)	36.0 (11.9)
Dietary intake, mean (s.d.)		
Total energy, kcal/day	2396 (839)	2164 (666)
Soy, g/day	88.4 (55.7)	79.8 (48.0)
Isoflavone, mg/day	33.1 (18.9)	30.2 (17.9)
The first urines, *n*	1106	223
Daidzein, mean (s.d.), µg/mL	6.1 (6.1)	5.0 (4.6)
Genistein, mean (s.d.), µg/mL	3.9 (4.2)	3.1 (3.0)
Glycitein, mean (s.d.), µg/mL	0.7 (0.7)	0.6 (0.5)
Equol, mean (s.d.), µg/mL^a^	0.5 (1.3)	0.3 (1.1)
Equol production, *n* (%)^b^		
(+)	288 (26.2%)	53 (23.9%)
(–)	812 (73.8%)	169 (76.1%)
Creatinine, mean (s.d.), mg/dL	127 (58)	122 (54)
The second urines, *n*	972	133
Daidzein, mean (s.d.), µg/mL	11.3 (8.5)	10.7 (6.6)
Genistein, mean (s.d.), µg/mL	8.2 (6.7)	7.3 (5.1)
Glycitein, mean (s.d.), µg/mL	0.9 (0.9)	0.8 (0.8)
Equol, mean (s.d.), µg/mL^a^	0.9 (2.4)	1.0 (2.8)
Equol production, *n* (%)^b^		
(+)	217 (22.3%)	28 (21.0%)
(-)	755 (77.7%)	105 (79.0%)
Creatinine, mean (s.d.), mg/dL	127 (59)	143 (60)

Dietary intakes indicate habitual intake during the past 6 months assessed by the food frequency questionnaire

The first and second urines were collected before and after two days of soymilk supplementation, respectively

*s*.*d*. standard deviation

^a^Means of urinary equol above the quantification limit (984 for the first urine samples and 904 for the second urine sample)

^b^The equol production positive was defined using a log-transformed equol/daidzein ratio of −1.75 or more

The proportion of equol production positive was little affected by sex and age, but it was relatively low in the youngest age group (7–8 years old) (Supplementary Table 2). The intakes of soy products and isoflavone were not associated with equol production. Urinary levels of daidzein and genistein were significantly lower in equol producers than in non-producers.

### The gut microbiota composition associated with equol production

**Fig. 1:**
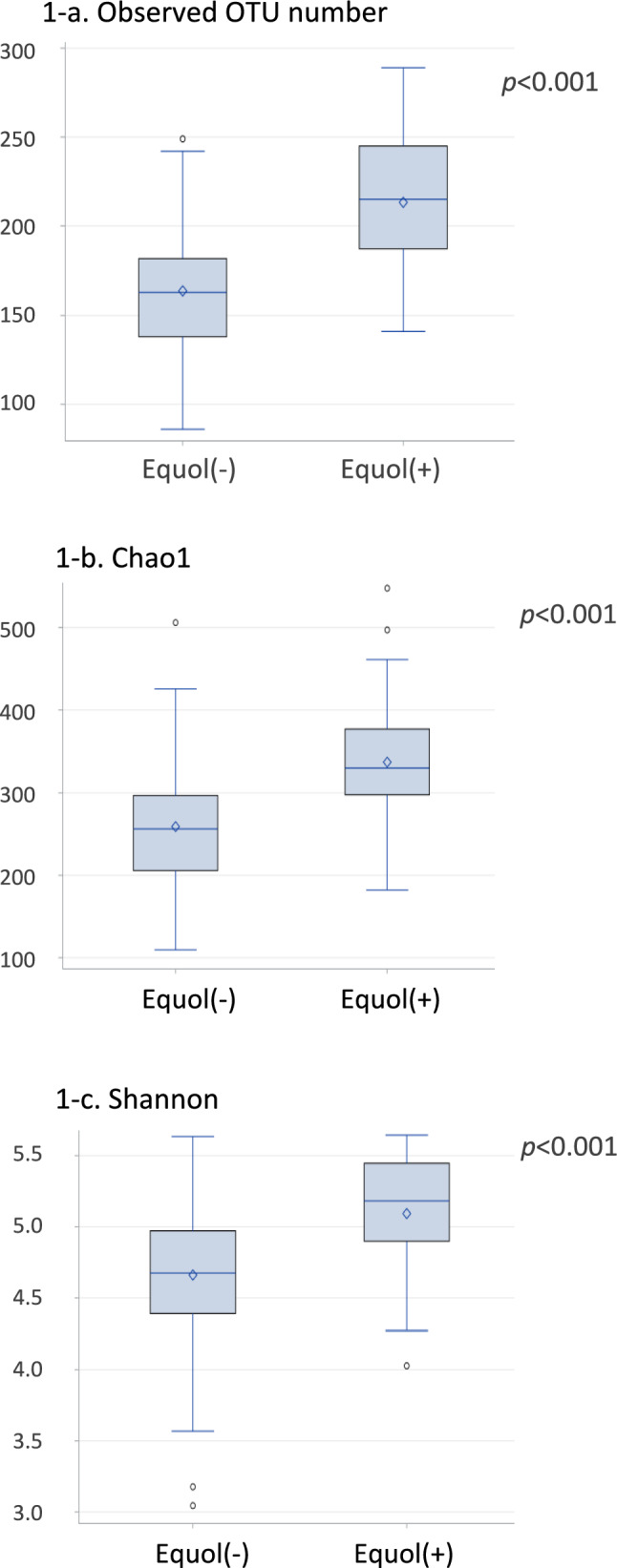
Comparison of gut microbiota α diversity between equol production (n=28) and non-production (n=105) in the second collected urine samples by analysis of covariance with adjustment for survey time, sex, and age. Equol production was determined in urine collected after two days of soymilk supplementation

In the gut microbiota of 223 participants, clustering of all high-quality 16S reads generated a total of 5,194 unique OTUs. The observed OTU number, Chao 1, and Shannon index were not affected by sex (Supplementary Figure 2). Chao 1 was significantly lower in the youngest age group (7–8 years old) than the older age groups (10–11 and 13–14 years old) (*p*=0.001). Isoflavone intake was not associated with the alpha-diversity indices (Supplementary Figure 3). All the three indices were higher in equol producers than in non-producers (Figure 1 and Supplementary Figure 4). Chao 1 was higher in equol producers than in non-producers in each of the three age groups (Supplementary Figure 5).

The positive associations between equol production and the three alpha-diversity indices were not altered by additional adjustment for isoflavone intake (all *p*<0.001). In addition, the indices of equol producers remained higher than those of non-producers after further adjustment for intakes of polyunsaturated fatty acids, vitamin A, vitamin E, and total cholesterol (all *p*<0.001).

**Fig. 2:**
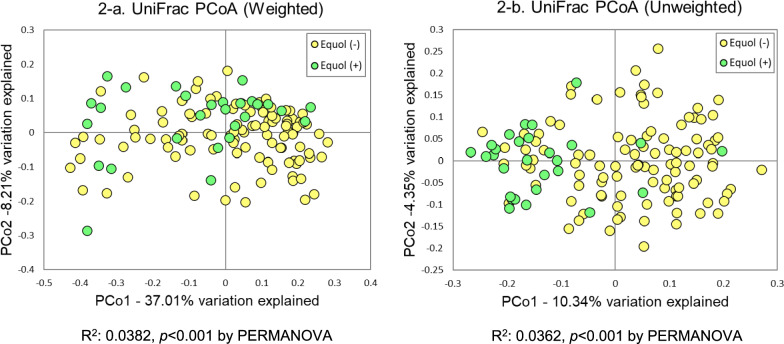
Principal coordinate analysis (PCoA) plots based on UniFrac distances of the gut microbiota for the comparison between equol production (n=28) and non-production (n=105) in the second collected urine samples. Equol production was determined in urine collected after two days of soymilk supplementation

In the analysis of beta-diversity, the PCoA plots based on weighted and unweighted UniFrac distances showed significant differences in the gut microbiota between equol producers and non-producers by PERMANOVA (Figure 2 and Supplementary Figure 6). Sex and age group did not modify the association between equol production and gut microbiota beta-diversity (Supplementary Figure 7 and 8). The isoflavone intake was not associated with beta-diversity of the gut microbiota (Supplementary Figure 9).

### The bacterial species associated with equol production

**Fig. 3:**
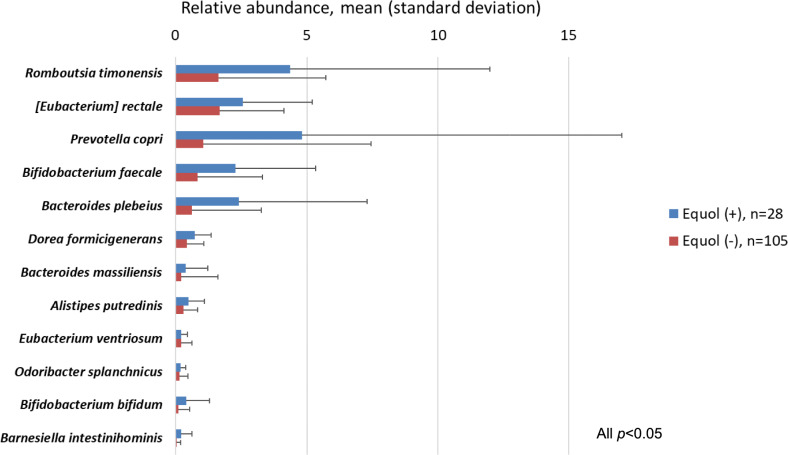
Twelve bacterial species with a mean relative abundance of >0.1% and with a significantly higher abundance in equol production than non-production in the second collected urine samples. Equol production was determined in urine collected after two days of soymilk supplementation. Relative abundances were compared between equol producers and non-producers using the Wilcoxon rank sum test with Benjamin-Hochberg adjustment

**Fig. 4:**
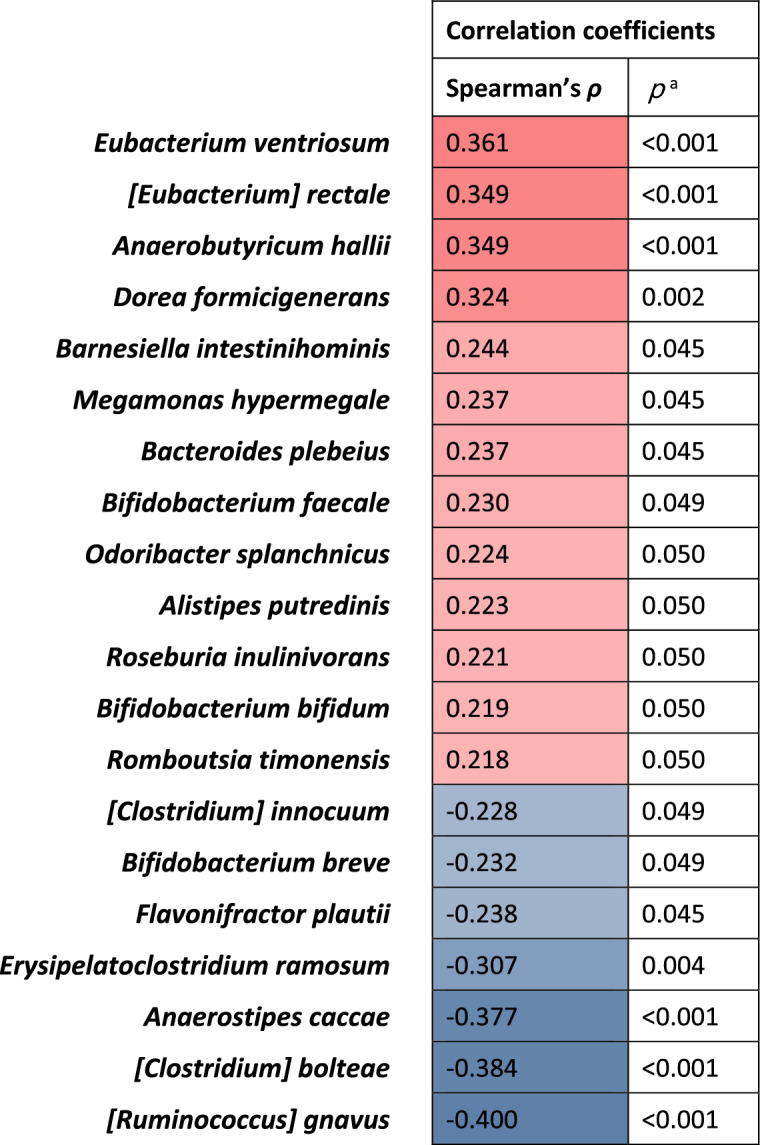
Heatmap of 20 species with a mean relative abundance of >0.1% and with a significant correlation with the equol/daidzein ratio in the second collected urine samples. Spearman’s correlation coefficients between the relative abundances of species and the equol/daidzein ratio in the urines collected after two days of soymilk supplementation (*n*=126). ^a^Benjamin-Hochberg adjusted *p* values

In the gut microbiota of 223 participants, 10 phyla, 209 genera, and 460 species were identified. Of the four phyla with an average relative abundance of ≥0.2%, *Firmicutes* was significantly more abundant and *Bacteroidetes* was less abundant in equol producers than non-producers (Supplementary Table 3). Of 34 genera with an average abundance of ≥0.2%, 7 genera had significantly higher relative abundance and 5 genera had lower abundance in equol producers than non-producers (Supplementary Table 4). In the species-level analysis, of 71 species with an average abundance of ≥0.1%, 12 species were significantly more abundant and 7 species were less abundant in equol producers than non-producers (Figure 3 and Supplementary Table 5). In addition, the correlation analysis found that 13 species were significantly positively and 7 species were inversely associated with the quol/daidzein ratio in the second urine samples (Figure 4 and Supplementary Table 6).

Of the 12 species previously reported in the literature as equol-producing, only three species (*Bifidobacterium longum, Bacteroides ovatus, Bifidobacterium breve*) had an average abundance of ≥0.1% in the participants. The detection rate ranged from 1.4 to 97.3% (Table 2 and Supplementary Table 7). *Asaccharobacter celatus* and *Slackia isoflavoniconvertens* were higher in relative abundance in equol producers than non-producers. *Bifidobacterium breve* was less abundant in equol producers than non-producers. The correlation analysis also revealed that the quol/daidzein ratio was positively associated with *Asaccharobacter celatus* and *Slackia isoflavoniconvertens* (Spearman’s ρ in the second urine was 0.63 (*p*<0.001) and 0.23 (*p*=0.010), respectively), and inversely associated with *Bifidobacterium breve* (Spearman’s ρ in the second urine: −0.23, *p*=0.009).

**Table 2:** Relative abundances of 12 potentially equol-producing species according to equol production in the second collected urine samples

Species	Detection rate (%)	Equol (+), *n*=28		Equol (–),* n*=105		
		mean	s.d.	mean	s.d.	*p*^a^
*Bifidobacterium longum*	97.3	2.564	2.289	3.811	3.631	0.184
*Bacteroides ovatus*	77.6	0.784	1.403	1.773	2.299	0.060
*Bifidobacterium breve*	52.9	**0.047**	**0.151**	**0.388**	**1.198**	**0.039**
*Lacticaseibacillus paracasei*	19.7	0.060	0.160	0.059	0.176	0.561
*Limosilactobacillus mucosae*	4.0	0.051	0.192	0.052	0.363	0.613
*Asaccharobacter celatus*	26.0	**0.061**	**0.074**	**0.004**	**0.012**	**<0.001**
*Latilactobacillus sakei*	1.4	0.000	0.000	0.020	0.203	0.373
*Slackia isoflavoniconvertens*	2.2	**0.016**	**0.060**	**0.000**	**0.000**	**<0.001**
*Streptococcus intermedius*	16.6	0.002	0.005	0.002	0.005	0.683
*Finegoldia magna*	8.1	0.001	0.004	0.003	0.015	0.773
*Lactococcus garvieae*	1.4	0.000	0.000	0.003	0.032	0.472
*Proteus mirabilis*	2.2	0.000	0.002	0.001	0.005	0.859

Equol production was determined in urine collected after two days of soymilk supplementation

*s*.*d*. standard deviation

^a^Relative abundances were compared between equol producers and non-producers using the Wilcoxon rank sum test

## Discussion

This study evaluated the association between equol production and the gut microbiota in children, revealing significant dissimilarities in the gut microbiota composition (alpha- and beta-diversity) between equol producers and non-producers. The differences were not affected by sex, age and isoflavone intake.

Eight previous studies have reported associations between equol production and the gut microbiome in humans [15, 16, 30–35]. However, equol production was assessed in different ways in these studies, including the measurement of isoflavones using LC-MS/MS [35], LC-MS [32], GC-MS [31], and HPLC [16, 30, 33, 34], the use of an immunochromatographic strip test [15], the assessment without dietary soy supplementation [15, 16, 31, 35], and the assessment without considering daidzein concentration [15, 30, 31, 34]. In addition, four Western studies had small sample sizes [30–33], and two of them did not evaluate the gut microbiota composition because of the use of T-RFLP [32, 33]. The other Asian studies had moderate sample sizes, and analyzed the gut microbiomes using 16S rRNA gene sequencing [15, 16, 35] or shotgun metagenomics sequencing [34]. The two Japanese studies reported a higher alpha-diversity in equol producers than in non-producers [15, 16], consistent with the present finding. Regarding beta diversity, three other studies besides this study reported significant differences in weighted UniFrac distances [30, 33] and Bray–Curtis distances [34] between equol producers and non-producers. Therefore, the microbiota composition may differ significantly between equol producers and non-producers, and the richness and evenness of a diverse microbiota may be important for equol production.

Among the bacterial species with an average abundance of ≥0.1%, we identified 15 species associated with equol production or the equol/daidzein ratio. However, these species were not previously reported as equol-producing microbes and did not belong to any characteristic types of bacteria with highly homologous sequences. A Chinese study found differences in the relative abundances of 32 species between equol producers and non-producers, including greater abundances of *Adlercreutzia equolifaciens* and *Bifidobacterium bifidum* in equol producers [34]. *Adlercreutzia equolifaciens* has been reported to produce equol [36], but was not detected in our fecal samples. *Bifidobacterium bifidum* showed a positive association with equol production in this study, too. *Bifidobacteria* are reported to convert daidzin to daidzein, but not to produce equol [37]. In order to assess whether the 15 identified species are involved in the metabolism from daidzein to equol, we used the KEGG Orthology database to identify genes coding for daidzein reductase (K26069), dihydrodaidzein reductase (K26070), tetrahydrodaidzein reductase (K26071), and dihydrodaidzein racemase (K26175), and then used Protein BLAST^®^ to check whether these gene codes were present in the genomes of the 15 species (identity > 30%, query coverage > 60%, e-value < 1×10^−5^). *Bifidobacterium bifidum*, *Anaerobutyricum hallii*, *Bifidobacterium faecale*, and *Romboutsia timonensis* had gene coding for daidzein reductase, and *Alistipes putredinis*, *Anaerobutyricum hallii*, *Roseburia inulinivorans*, *[Eubacterium] rectale*, *Dorea formicigenerans*, *Romboutsia timonensis*, *Megamonas hypermegale*, and *Odoribacter splanchnicus* had gene coding for dihydrodaidzein reductase. However, the other five species did not have the gene codes for these enzymes. There were no species having gene codes for dihydrodaidzein racemase and tetrahydrodaidzein reductase.

Most of the equol-producing species reported in the literature had an average abundance of less than 0.1%. *Asaccharobacter celatus* and *Slackia isoflavoniconvertens* had a higher relative abundance among equol producers compared with non-producers, consistent with a previous Japanese study [16]. Although these two species have been shown to be capable of producing equol from daidzein in experimental studies [38, 39], the average abundances of *Asaccharobacter celatus* and *Slackia isoflavoniconvertens* were 0.014% and 0.003%, respectively, suggesting that these species alone are unlikely to have a major impact on the overall microbiota composition. On the other hand, another potentially equol-producing species, *Bifidobacterium breve,* tended to be less abundant in equol producers than non-producers. The reason for this was unclear, but individual strains of the same species may have different effects on equol production. Most previous experimental studies on microbes involved in equol production have identified isolated strains, but not at the species level. It could also suggest that the biological effects of microbes are influenced by other microbes living symbiotically in the gut. The wide variation of species listed in this study as having equol-producing potential suggests that the biosynthesis of equol may involve enzyme genes with diverse and general activities, such as hydrolase and reductase, regardless of bacterial classification.

This study found no significant association between soy isoflavone intake and equol production, which is consistent with previous Asian studies [14, 34, 40–44]. Higher urinary daidzein levels in equol non-producers may reflect the fact that participants who cannot produce equol would not produce equol no matter how much isoflavone they consumed. However, the prevalence of equol producers has been reported to be higher in Asians or in vegetarians who consume more soy than in people who eat a typical Western diet [28, 42]. Equol production has been associated with higher soy intake in Caucasian adult men [45]. Children fed a soy-based infant formula had a higher prevalence of equol production at 4–6 months of age than those fed a dairy milk formula, but not at older ages [46]. Thus, the effect of soy on equol production might be limited to a lower range of consumption and/or a relatively young age. Whether dietary components other than soy may promote the ability to produce equol remains unknown [28, 34, 40–42, 44, 45, 47]. Further studies focusing on various other components are needed to elucidate which dietary habits influence equol production.

A major strength of this study is the diagnosis of equol production after soymilk supplementation, thereby accurately evaluating the ability to produce equol. However, the positive rate of equol production did not increase significantly after soy supplementation, despite the increase in urinary isoflavone levels reflecting the success of soy supplementation. This may indicate that many of the participating children routinely ate enough soy foods to assess their ability to produce equol. Other strengths include the relatively large sample size, high participation rate, and use of a validated FFQ to measure habitual dietary intake. Nevertheless, some potential limitations should be addressed. First, the correlation coefficients between FFQ and the diet records were not so high, but they were similar to those of other nutritional epidemiological studies in children [22]. Some degree of measurement error in dietary intake, which would have non-differentially occurred regardless of equol status, might have led to a null association between soy isoflavone intake and equol-producing status. Second, because this is a cross-sectional study, we cannot infer causality. However, it is unlikely that equol-producing status would alter dietary intake or microbial composition because equol is a microbial metabolite. Next, the 16S rRNA gene sequencing analysis might not have provided sufficient taxonomic resolution to conduct a species-level association study. Whole-genome metagenomic analysis would be desirable in the future. The children studied in this study were Japanese and consumed a much higher amount of soy foods compared with Westerners, which may affect the generalizability of our findings. Lastly, although some of the associations between equol production and microbiota might have occurred by chance, the microbiota composition listed in this study will inform future studies to determine the interrelationships among microbes involved in equol production.

## Conclusion

In conclusion, this study of Japanese school children aged 7–14 years identified several new microbes which may be involved in equol production. The species *Asaccharobacter celatus* and *Slackia isoflavoniconvertens* may play important roles in equol production in the human gut. The richness of a diverse microbiota and the interrelationships among microbes may be important for equol production.

## Supplementary Information

Below is the link to the electronic supplementary material.

Supplementary file1 (DOCX 1347 KB)

## Data Availability

The 16S rRNA gene V1-V2 region sequences analyzed in this study were deposited in DDBJ/GenBank/EMBL with the accession numbers DRA016182 (available upon publication). The other informative data described in the manuscript will be made available upon request.

## Abbreviations

ANCOVA: Analysis of covariance
FFQ: Food frequency questionnaire
LC–MS: Liquid chromatography–mass spectrometry
OUT: Operational taxonomic unit
PCoA: Principal coordinate analysis
PERMANOVA: Permutational multivariate analysis of variance
