# Supplementary material for: Gut microbiota associated with equol production in school-age children
Source: Eur J Nutr. 2025 May 9;64(4):174. doi: 10.1007/s00394-025-03625-w (PMC12064588; doi:10.1007/s00394-025-03625-w)
Supplement: Supplementary file 1 — Supplementary file1 (DOCX 1347 KB) [file 394_2025_3625_MOESM1_ESM.docx]

**Supplements**

**Supplementary Material 1. Measurement of urinary isoflavones and metabolites**

**Supplementary Material 2. Measurement of the gut microbiota**

**Supplementary Table 1. Dietary sources of isoflavones among 1,110 participants**

**Supplementary Table 2. Sex, age, soy isoflavone intakes, and urinary isoflavone levels according to equol production**

**Supplementary Table 3. Relative abundances of the four bacterial phyla with a mean abundance of >0.2% according to equol production**

**Supplementary Table 4. Relative abundance of the 34 bacterial genera with a mean relative abundance of >0.2% according to equol production**

**Supplementary Table 5. Relative abundance of the 71 bacterial species with a mean relative abundance of >0.1% according to equol production**

**Supplementary Table 6. Spearman’s correlation coefficients between the equol/daidzein ratio and the relative abundances of 71 bacterial species with a mean relative abundance of >0.1%**

**Supplementary Table 7. Relative abundances of 12 potentially equol-producing species according to equol production in the first urine samples.**

**Supplementary Figure 1. Flowchart of the study participants**

**Supplementary Figure 2. Indices of gut microbiota α-diversity according to sex and age group**

**Supplementary Figure 3. Indices of gut microbiota α-diversity according to tertiles of isoflavone intake**

**Supplementary Figure 4. Comparison of gut microbiota α-diversity between equol producers (n=53) and non-producers (n=169) in the first collected urine samples by analysis of covariance with adjustment for survey time, sex, and age**

**Supplementary Figure 5. Comparison of Chao1 between equol producers and non-producers by age group**

**Supplementary Figure 6. Principal coordinate analysis (PCoA) plots based on UniFrac distances of the gut microbiota for the comparison between equol production (n=53) and non-production (n=169) in the first urines**

**Supplementary Figure 7. Principal coordinate analysis (PCoA) plots based on UniFrac distances of the gut microbiota for the comparison between equol production and non-production by sex**

**Supplementary Figure 8. Principal coordinate analysis (PCoA) plots based on UniFrac distances of the gut microbiota for the comparison between equol producers and non-producers by age group**

**Supplementary Figure 9. Principal coordinate analysis (PCoA) plots based on UniFrac distances of the gut microbiota according to isoflavone intake (n=223)**

**Supplementary Material 1. Measurement of urinary isoflavones and metabolites**

Sample preparation was performed as reported by Fleck et al.,^1)^ with minor modifications. Samples were incubated for 1 h at 37°C for enzymatic hydrolysis. Sample clean-up was performed using Isolute SLE+ 96-well plates (Biotage, Uppsala, Sweden). The eluent was evaporated for dryness under nitrogen and then reconstituted with 50% methanol (500 μL).

Analyses were performed using the Nexera X2 UHPLC system (Shimadzu, Kyoto, Japan) coupled with a Triple Quad 5500+ mass spectrometer (AB Sciex LLC, Framingham, MA) with an atmospheric pressure chemical ionization (APCI) ion source, negative-ion mode. The APCI source parameters were set as follows: collision-activated dissociation gas (N_2_) at 8 psig, curtain gas at 20 psig, ion source gas at 40 psig, heated nebulizer temperature at 400°C, and discharge needle current at −2 μA. The multiple reaction monitoring transitions monitored were as follows: 241→121 for equol, 246→94 for equol-d4, 253→223 for daidzein, 256→137 for daidzein-d6, 269→133 for genistein, and 274→137 for genistein-d4.

For urine analysis, a Sunshell C18 column (2.1 × 100 mm, 2.6 μm; ChromaNik Technologies Inc., Osaka, Japan) was used at 40°C. The injection volume was 1 μL. Mobile phase A consisted of a 0.25-mmol/L ammonium acetate buffer (pH 6)/methanol (6/4, v/v), and mobile phase B was acetonitrile. The flow rate was set to 0.3 mL/min. The gradient was as follows: 20% B for 2 min, from 20% to 45% B in 1.5 min, 45% to 95% B in 0.01 min, 95% B for 4.5 min, from 95% to 20% B in 0.01 min, and then 20% B for 1 min.

1) Fleck SC, Churchwell MI, Doerge DR, Teeguarden JG, et al. Urine and serum biomonitoring of exposure to environmental estrogens II: Soy isoflavones and zearalenone in pregnant women. *Food Chem Toxicol.* 2016;95:19-27.

**Supplementary Material 2.** **Measurement of the gut microbiota**

**DNA extraction and 16S amplicon sequencing**

Fecal DNA was isolated and purified following a previously described method.^2)^ The DNA was purified with a 20% PEG solution (PEG6000 in 2.5 M NaCl), pelleted by centrifugation, rinsed with 75% ethanol, and dissolved in TE buffer. The 16S ribosomal RNA gene V1-V2 region was amplified by polymerase chain reaction (PCR) using the forward primer (5′-AATGATACGGCGACCACCGAGATCTACAC NNNNNNNNACACTCTTTCCCTACACGACGCTCTTCCGATCTagrgtttgatymtggctcag-3′) containing the flow cell adapter (Illumina Inc., San Diego, CA), a unique 8-bp barcode sequence for each sample (indicated in N), the sequence adapter (Illumina), and 27Fmod (5′-agrgtttgatymtggctcag),^2)^ and the reverse primer (5′-CAAGCAGAAGACGGCATACGAGATNNNNNNNNGTGACTGGAGT TCAGACGTGTG CTCTTCCGATCTtgctgcctcccgtaggagt-3′ ) containing the flow cell adapter, a unique 8-bp barcode sequence for each sample (indicated in N), the sequence adapter, and 338R (5′- tgctgcctcccgtaggagt-3′). PCR was performed in 50 μL of 1× Ex Taq PCR buffer composed of 10 mM Tris–HCl (pH 8.3), 50 mM KCl, and 1.5 mM MgCl2 in the presence of 250 mM dNTP, 1 U Ex Taq polymerase (Takara Bio, Inc.), forward and reverse primers (0.2 mM) and ~20 ng template DNA. Thermal cycling consisted of initial denaturation at 96°C for 2 min, followed by 20 cycles of denaturation at 96°C for 30 s, annealing at 55°C for 45 s and extension at 72°C for 1 min, and final extension at 72°C on a PCR system (Applied Biosystems, Foster City, CA). Negative controls were treated similarly, except that no template DNA was added to the PCR reactions. PCR products of ~370 bp were visualized by electrophoresis on 3% agarose gels, while negative controls failed to produce visible PCR products and were excluded from further analysis. PCR amplicons were purified by AMPure XP magnetic purification beads (Beckman Coulter, Inc., Brea, CA), and quantified using the Quant-iT PicoGreen dsDNA Assay Kit (Life Technologies, Carlsbad, CA). Equal amounts of each PCR amplicon were mixed and sequenced with the MiSeq Platform using MiSeq Reagent Kit v3 (600 cycles; Illumina).

**Data analysis**

After demultiplexing the 16S sequence reads based on the sample-specific index, the reads lacking both forward and reverse primer sequences were removed using BLAST and Cutadapt version 1.15 followed by trimming off both primer sequences. Data were further denoised by removing reads with average quality values <25. Reads having BLAST match lengths <90% with the representative sequence in the 16S databases were considered as chimeras and removed. Finally, filter-passed reads were used for further analysis.

2) Kim SW, Suda W, Kim S. et al. Robustness of gut microbiota of healthy adults in response to probiotic intervention revealed by high-throughput pyrosequencing. *DNA Res*. 2013;20:241-253.

| **Supplementary Table 1.** Dietary sources of isoflavones among 1,110 participants | |
| --- | --- |
|  | percentages |
| Soybeans | 3.1% |
| Soybeans, green | 3.9% |
| Kinako (roasted and ground soybeans) | 0.1% |
| Tofu | 44.6% |
| Natto (fermented soybeans) | 14.9% |
| Soymilk | 4.6% |
| Soybean sprouts | 4.9% |
| Miso | 22.2% |
| Soy sauce | 1.2% |
| Soy snacks | 0.3% |

| **Supplementary Table 2.** Sex, age, soy isoflavone intakes, and urinary isoflavone levels according to equol production | | | | | | | | | | | | | | | | | | | | | | | | | | | | | | | | | |
| --- | --- | --- | --- | --- | --- | --- | --- | --- | --- | --- | --- | --- | --- | --- | --- | --- | --- | --- | --- | --- | --- | --- | --- | --- | --- | --- | --- | --- | --- | --- | --- | --- | --- |
|  | The first collected urine samples | | | | | | | | |  | |  | | The second collected urine samples | | | | | | | | | | | | | | | | | |  | |
|  | Equol (+), *n*=288 | | | |  | Equol (-), *n*=812 | | | |  | |  | | Equol (+), *n*=217 | | | | | | | |  | | Equol (-), *n*=755 | | | | | | | |  | |
|  | n | % | estimated mean | s.e. |  | n | % | estimated mean | s.e. | *p*^a^ | |  | | n | | % | | estimated mean | | s.e. | |  | | n | | % | | estimated mean | | s.e. | | *p*^a^ | |
| Sex |  |  |  |  |  |  |  |  |  |  | |  | |  | |  | |  | |  | |  | |  | |  | |  | |  | |  | |
| boy | 140 | 25.2% |  |  |  | 415 | 74.8% |  |  | 0.47 | |  | | 111 | | 22.8% | |  | |  | |  | | 376 | | 77.2% | |  | |  | | 0.73 | |
| girl | 148 | 27.2% |  |  |  | 397 | 72.8% |  |  |  | |  | | 106 | | 21.9% | |  | |  | |  | | 379 | | 78.1% | |  | |  | |  | |
| Age |  |  |  |  |  |  |  |  |  |  | |  | |  | |  | |  | |  | |  | |  | |  | |  | |  | |  | |
| 7-8 years old | 66 | 24.6% |  |  |  | 202 | 75.4% |  |  | 0.74 | |  | | 29 | | 17.6% | |  | |  | |  | | 136 | | 82.4% | |  | |  | | 0.27 | |
| 10-11 years old | 79 | 27.5% |  |  |  | 208 | 72.5% |  |  |  |  | | 62 | | 23.2% | |  | |  | |  | | 205 | | 76.8% | |  | |  | |  | |  |
| 13-14 years old | 143 | 26.2% |  |  |  | 402 | 73.8% |  |  |  | |  | | 126 | | 23.3% | |  | |  | |  | | 414 | | 76.7% | |  | |  | |  | |
| Daily intake |  |  |  |  |  |  |  |  |  |  | |  | |  | |  | |  | |  | |  | |  | |  | |  | |  | |  | |
| soy (g) |  |  | 86.0 | 2.9 |  |  |  | 85.7 | 1.7 | 0.94 | |  | |  | |  | | 89.0 | | 3.4 | |  | |  | |  | | 84.1 | | 1.8 | | 0.20 | |
| isoflavone (mg) |  |  | 32.9 | 1.0 |  |  |  | 33.2 | 0.6 | 0.76 | |  | |  | |  | | 34.0 | | 1.1 | |  | |  | |  | | 32.8 | | 0.6 | | 0.43 | |
| Urinary level  (µg/g creatinine) |  |  |  |  |  |  |  |  |  |  | |  | |  | |  | |  | |  | |  | |  | |  | |  | |  | |  | |
| daidzein |  |  | 3.7 | 0.3 |  |  |  | 6.1 | 0.2 | <0.001 | |  | |  | |  | | 7.4 | | 0.5 | |  | |  | |  | | 10.6 | | 0.3 | | <0.001 | |
| genistein |  |  | 2.2 | 0.2 |  |  |  | 3.9 | 0.1 | <0.001 | |  | |  | |  | | 4.8 | | 0.4 | |  | |  | |  | | 7.9 | | 0.2 | | <0.001 | |
| glycitein |  |  | 0.5 | 0.04 |  |  |  | 0.7 | 0.02 | 0.003 | |  | |  | |  | | 0.7 | | 0.06 | |  | |  | |  | | 0.8 | | 0.03 | | 0.25 | |
| s.e.: standard error. Soy and isoflavone intakes indicate habitual intake during the past 6 months assessed by the food frequency questionnaire, which are adjusted for total energy by using the residual method proposed by Willett. The first and second urines were collected before and after two days of soymilk supplementation, respectively. | | | | | | | | | | | | | | | | | | | | | | | | | | | | | | | | | |
| ^a^*p* values for the comparison by *χ^2^* test (for sex and age) or analysis of covariance with adjustment for survey time, sex, and age (for soy isoflavone intakes and urinary isoflavone levels). | | | | | | | | | | | | | | | | | | | | | | | | | | | | | | | | | |

| **Supplementary Table 3.** Relative abundances of the four bacterial phyla with a mean abundance of >0.2% according to equol production | | | | | | | | | | | | | | | | | |
| --- | --- | --- | --- | --- | --- | --- | --- | --- | --- | --- | --- | --- | --- | --- | --- | --- | --- |
|  | The first collected urine samples | | | | | | |  |  | The second collected urine samples | | | | | | |  |
|  | Equol (+), *n*=53 | |  | Equol (-), *n*=169 | |  | *p*^a^ |  |  | Equol (+), *n*=28 | |  | Equol (-), *n*=105 | |  | *p*^a^ |  |
|  | mean | s.d. |  | mean | s.d. |  |  |  |  | mean | s.d. |  | mean | s.d. |  |  |  |
| ***Firmicutes*** | 55.44 | 12.61 |  | 54.04 | 13.64 |  | 0.915 |  |  | **57.74** | **12.51** |  | **50.06** | **12.77** |  | **0.023** | ***** |
| ***Bacteroidetes*** | 32.16 | 15.70 |  | 32.65 | 14.63 |  | 0.960 |  |  | **27.42** | **16.50** |  | **35.07** | **15.18** |  | **0.037** | ***** |
| ***Actinobacteria*** | 10.63 | 7.51 |  | 11.16 | 9.01 |  | 0.960 |  |  | 12.67 | 8.50 |  | 12.50 | 10.03 |  | 0.540 |  |
| ***Proteobacteria*** | 1.68 | 1.29 |  | 2.01 | 1.57 |  | 0.315 |  |  | 2.04 | 1.60 |  | 2.19 | 1.75 |  | 0.540 |  |
| s.d.: standard deviation. The first and second urines were collected before and after two days of soymilk supplementation, respectively.  ^a^Relative abundances were compared between equol producers and non-producers using the Wilcoxon rank sum test with Benjamin-Hochberg adjustment. *p<0.05, **p<0.01, ***p<0.001.  Orange asterisks indicate a higher abundance of the phylum in equol producers. Blue asterisks indicate a lower abundance of the phylum in equol producers. | | | | | | | | | | | | | | | | | |

| **Supplementary Table 4.** Relative abundance of the 34 bacterial genera with a mean relative abundance of >0.2% according to equol production | | | | | | | | | | | | | | | |
| --- | --- | --- | --- | --- | --- | --- | --- | --- | --- | --- | --- | --- | --- | --- | --- |
|  | The first collected urine samples | | | | | |  |  | The second collected urine samples | | | | | |  |
|  | Equol (+), *n*=53 | |  | Equol (-), *n*=169 | | *p*^a^ |  |  | Equol (+), *n*=28 | |  | Equol (-), *n*=105 | | *p*^a^ |  |
|  | mean | s.d. |  | mean | s.d. |  |  |  | mean | s.d. |  | mean | s.d. |  |  |
| ***Bacteroides*** | 22.78 | 13.15 |  | 26.22 | 13.42 | 0.199 |  |  | **18.27** | **11.39** |  | **28.62** | **14.20** | **0.006** | ****** |
| ***Blautia*** | 11.28 | 4.25 |  | 13.15 | 5.86 | 0.125 |  |  | 11.53 | 4.39 |  | 12.47 | 5.84 | 0.763 |  |
| ***Bifidobacterium*** | 9.18 | 7.34 |  | 10.20 | 8.67 | 0.623 |  |  | 11.04 | 8.35 |  | 11.39 | 9.58 | 0.996 |  |
| ***Faecalibacterium*** | 9.13 | 4.56 |  | 7.85 | 5.54 | 0.125 |  |  | 7.81 | 4.50 |  | 7.16 | 4.98 | 0.578 |  |
| ***Fusicatenibacter*** | 3.52 | 2.56 |  | 4.65 | 4.08 | 0.245 |  |  | 4.13 | 3.84 |  | 4.65 | 4.20 | 0.514 |  |
| ***Anaerostipes*** | 4.37 | 4.11 |  | 4.28 | 3.70 | 0.953 |  |  | 4.01 | 4.42 |  | 3.65 | 3.43 | 0.763 |  |
| ***Streptococcus*** | 2.17 | 3.48 |  | 2.58 | 3.10 | 0.489 |  |  | 3.02 | 4.77 |  | 2.39 | 2.99 | 0.763 |  |
| ***Romboutsia*** | 3.09 | 5.91 |  | 2.20 | 4.85 | 0.125 |  |  | **4.37** | **7.66** |  | **1.66** | **4.11** | **0.024** | ***** |
| ***G_undefined_Lachnospiraceae*** | 2.53 | 2.98 |  | 2.35 | 3.17 | 0.403 |  |  | **2.59** | **2.65** |  | **1.70** | **2.49** | **0.030** | ***** |
| ***Parabacteroides*** | 1.94 | 1.48 |  | 2.47 | 2.41 | 0.656 |  |  | 1.54 | 1.24 |  | 2.61 | 2.37 | 0.129 |  |
| ***Prevotella*** | 4.68 | 11.13 |  | 1.12 | 6.36 | 0.064 |  |  | **5.03** | **12.60** |  | **1.26** | **7.38** | **0.005** | ****** |
| ***Anaerobutyricum*** | 2.21 | 2.10 |  | 1.85 | 1.91 | 0.429 |  |  | **2.85** | **2.16** |  | **1.77** | **2.05** | **0.024** | ***** |
| ***Roseburia*** | 1.31 | 1.66 |  | 1.17 | 2.03 | 0.149 |  |  | 1.20 | 1.87 |  | 1.00 | 2.11 | 0.385 |  |
| ***Intestinibacter*** | 0.73 | 0.73 |  | 0.98 | 1.14 | 0.613 |  |  | 0.90 | 1.03 |  | 0.88 | 0.97 | 0.808 |  |
| ***Faecalicatena*** | 0.95 | 1.14 |  | 0.86 | 1.28 | 0.144 |  |  | 1.22 | 1.31 |  | 0.91 | 1.34 | 0.130 |  |
| ***Robinsoniella*** | **0.41** | **0.64** |  | **0.99** | **1.35** | **0.011** | ***** |  | **0.24** | **0.45** |  | **1.00** | **1.33** | **0.016** | ***** |
| ***Collinsella*** | 1.16 | 1.74 |  | 0.72 | 1.38 | 0.099 |  |  | 1.28 | 1.84 |  | 0.84 | 1.64 | 0.092 |  |
| ***Lachnoclostridium*** | 0.64 | 0.50 |  | 0.84 | 0.64 | 0.113 |  |  | 0.61 | 0.51 |  | 0.88 | 0.68 | 0.076 |  |
| ***Alistipes*** | **1.21** | **1.53** |  | **0.62** | **1.00** | **<0.001** | ******* |  | **1.29** | **1.59** |  | **0.56** | **0.83** | **0.005** | ****** |
| ***Veillonella*** | **0.39** | **0.82** |  | **0.85** | **1.26** | **0.008** | ****** |  | **0.38** | **0.90** |  | **0.90** | **1.44** | **0.047** | ***** |
| ***Eubacterium*** | 0.64 | 0.50 |  | 0.60 | 0.70 | 0.190 |  |  | **0.66** | **0.45** |  | **0.53** | **0.71** | **0.034** | ***** |
| ***Parasutterella*** | 0.39 | 0.55 |  | 0.64 | 0.92 | 0.798 |  |  | 0.54 | 0.67 |  | 0.62 | 0.94 | 0.284 |  |
| ***Sutterella*** | 0.57 | 0.73 |  | 0.52 | 0.84 | 0.429 |  |  | 0.57 | 0.76 |  | 0.55 | 0.84 | 0.763 |  |
| ***Drancourtella*** | 0.47 | 0.77 |  | 0.50 | 0.52 | 0.232 |  |  | **0.34** | **0.89** |  | **0.48** | **0.53** | **0.024** | ***** |
| ***Dorea*** | **0.62** | **0.60** |  | **0.37** | **0.59** | **0.003** | ****** |  | **0.75** | **0.62** |  | **0.44** | **0.65** | **0.010** | ***** |
| ***Ruminococcus*** | **0.47** | **0.66** |  | **0.38** | **0.88** | **0.004** | ****** |  | 0.38 | 0.56 |  | 0.30 | 0.72 | 0.080 |  |
| ***Megamonas*** | **0.60** | **1.71** |  | **0.35** | **2.02** | **0.010** | ****** |  | 0.69 | 1.93 |  | 0.46 | 2.44 | 0.204 |  |
| ***Clostridium*** | 0.26 | 0.42 |  | 0.45 | 1.18 | 0.798 |  |  | 0.22 | 0.28 |  | 0.26 | 0.59 | 0.385 |  |
| ***Butyricicoccus*** | 0.41 | 0.33 |  | 0.39 | 0.32 | 0.645 |  |  | 0.41 | 0.39 |  | 0.43 | 0.34 | 0.763 |  |
| ***Erysipelatoclostridium*** | **0.23** | **0.38** |  | **0.45** | **0.54** | **<0.001** | ******* |  | **0.19** | **0.31** |  | **0.49** | **0.58** | **0.002** | ****** |
| ***Escherichia*** | 0.25 | 0.63 |  | 0.41 | 1.04 | 0.104 |  |  | 0.40 | 0.95 |  | 0.49 | 1.13 | 0.717 |  |
| ***Lachnospira*** | 0.30 | 0.46 |  | 0.39 | 0.71 | 0.904 |  |  | 0.17 | 0.26 |  | 0.29 | 0.56 | 0.763 |  |
| ***Turicibacter*** | 0.27 | 0.42 |  | 0.33 | 0.77 | 0.645 |  |  | 0.30 | 0.50 |  | 0.30 | 0.67 | 0.763 |  |
| ***Dialister*** | 0.36 | 0.54 |  | 0.20 | 0.35 | 0.356 |  |  | 0.34 | 0.46 |  | 0.20 | 0.33 | 0.554 |  |
| s.d.: standard deviation. The first and second urines were collected before and after two days of soymilk supplementation, respectively.  ^a^Relative abundances were compared between equol producers and non-producers using the Wilcoxon rank sum test with Benjamin-Hochberg adjustment.  **p*<0.05, ***p*<0.01, ****p*<0.001.  Orange asterisks indicate a higher abundance of the genus in equol producers.  Blue asterisks indicate a lower abundance of the genus in equol producers. | | | | | | | | | | | | | | | |

| **Supplementary Table 5.** Relative abundance of the 71 bacterial species with a mean relative abundance of >0.1% according to equol production | | | | | | | | | | | | | | | |
| --- | --- | --- | --- | --- | --- | --- | --- | --- | --- | --- | --- | --- | --- | --- | --- |
|  | The first collected urine samples | | | | | |  |  | The second collected urine samples | | | | | |  |
|  | Equol (+), *n*=53 | |  | Equol (-), *n*=169 | | *p^a^* |  |  | Equol (+), *n*=28 | |  | Equol (-), *n*=105 | | *p^a^* |  |
|  | mean | s.d. |  | mean | s.d. |  |  |  | mean | s.d. |  | mean | s.d. |  |  |
| ***Blautia luti*** | 6.15 | 3.15 |  | 7.37 | 4.50 | 0.194 |  |  | 6.51 | 2.71 |  | 6.76 | 4.45 | 0.955 |  |
| ***Bacteroides vulgatus*** | 4.90 | 6.83 |  | 7.59 | 9.66 | 0.604 |  |  | 4.96 | 5.29 |  | 7.47 | 10.30 | 0.860 |  |
| ***Bacteroides dorei*** | 3.57 | 5.10 |  | 5.56 | 7.71 | 0.717 |  |  | 1.52 | 2.22 |  | 6.31 | 7.80 | 0.090 |  |
| ***Bifidobacterium pseudocatenulatum*** | 3.81 | 4.49 |  | 4.59 | 5.61 | 0.899 |  |  | 3.44 | 5.22 |  | 5.12 | 6.34 | 0.583 |  |
| ***Faecalibacterium prausnitzii*** | **5.05** | **3.25** |  | **3.90** | **3.53** | **0.043** | ***** |  | 4.29 | 2.73 |  | 3.79 | 3.31 | 0.371 |  |
| ***Anaerostipes hadrus*** | 4.22 | 4.15 |  | 4.09 | 3.80 | 0.899 |  |  | 3.93 | 4.40 |  | 3.44 | 3.52 | 0.655 |  |
| ***Bifidobacterium longum*** | 2.34 | 2.02 |  | 3.31 | 3.34 | 0.286 |  |  | 2.56 | 2.29 |  | 3.81 | 3.63 | 0.319 |  |
| ***Fusicatenibacter saccharivorans*** | 2.34 | 1.75 |  | 3.24 | 3.03 | 0.232 |  |  | 2.69 | 2.21 |  | 3.28 | 3.19 | 0.500 |  |
| ***Bacteroides stercoris*** | 2.05 | 3.93 |  | 2.70 | 4.97 | 0.608 |  |  | 0.97 | 1.70 |  | 3.35 | 5.64 | 0.655 |  |
| ***Romboutsia timonensis*** | 3.07 | 5.89 |  | 2.19 | 4.83 | 0.123 |  |  | **4.36** | **7.63** |  | **1.64** | **4.08** | **0.030** | ***** |
| ***[Eubacterium] rectale*** | 2.51 | 2.95 |  | 2.33 | 3.14 | 0.377 |  |  | **2.57** | **2.63** |  | **1.68** | **2.45** | **0.034** | ***** |
| ***Bacteroides uniformis*** | 2.65 | 3.95 |  | 2.14 | 3.11 | 0.391 |  |  | 1.96 | 3.33 |  | 2.37 | 3.33 | 0.992 |  |
| ***Bacteroides ovatus*** | 1.01 | 1.87 |  | 1.88 | 2.67 | 0.198 |  |  | 0.78 | 1.40 |  | 1.77 | 2.30 | 0.151 |  |
| ***Prevotella copri*** | 3.64 | 10.13 |  | 0.89 | 5.46 | 0.232 |  |  | **4.82** | **12.20** |  | **1.06** | **6.38** | **0.018** | ***** |
| ***Bacteroides fragilis*** | **0.93** | **2.01** |  | **1.74** | **4.18** | **0.033** | ***** |  | **1.01** | **2.43** |  | **1.77** | **4.56** | **0.034** | ***** |
| ***[Ruminococcus] gnavus*** | **0.85** | **1.76** |  | **1.68** | **2.20** | **0.002** | ****** |  | **0.41** | **0.69** |  | **1.77** | **2.37** | **0.002** | ****** |
| ***Bacteroides coprocola*** | 1.23 | 3.48 |  | 0.85 | 3.44 | 0.072 |  |  | 0.94 | 2.68 |  | 1.17 | 4.15 | 0.153 |  |
| ***Bifidobacterium faecale*** | **1.28** | **2.42** |  | **0.76** | **2.13** | **0.043** | ***** |  | **2.30** | **3.03** |  | **0.84** | **2.48** | **0.009** | ****** |
| ***Bifidobacterium adolescentis*** | 1.20 | 2.59 |  | 0.74 | 2.04 | 0.351 |  |  | 1.89 | 3.32 |  | 0.79 | 2.24 | 0.204 |  |
| ***Streptococcus salivarius*** | 0.85 | 0.90 |  | 0.83 | 1.04 | 0.544 |  |  | 0.78 | 0.71 |  | 0.88 | 1.04 | 0.832 |  |
| ***Intestinibacter bartlettii*** | 0.66 | 0.67 |  | 0.87 | 1.03 | 0.604 |  |  | 0.81 | 0.93 |  | 0.78 | 0.86 | 0.832 |  |
| ***Bacteroides plebeius*** | **1.97** | **4.24** |  | **0.43** | **2.17** | **<0.001** | ******* |  | **2.42** | **4.88** |  | **0.62** | **2.64** | **0.011** | ***** |
| ***Streptococcus equinus*** | 0.39 | 1.10 |  | 0.87 | 2.47 | 0.365 |  |  | 0.89 | 2.18 |  | 0.85 | 2.60 | 0.947 |  |
| ***[Ruminococcus] torques*** | 0.80 | 1.60 |  | 0.73 | 1.07 | 0.861 |  |  | 0.87 | 1.74 |  | 0.73 | 1.00 | 0.704 |  |
| ***Streptococcus pasteurianus*** | 0.80 | 3.38 |  | 0.71 | 1.99 | 0.354 |  |  | 1.21 | 4.45 |  | 0.52 | 1.38 | 0.603 |  |
| ***Collinsella aerofaciens*** | 0.98 | 1.40 |  | 0.62 | 1.25 | 0.072 |  |  | 1.11 | 1.56 |  | 0.72 | 1.47 | 0.168 |  |
| ***Parabacteroides merdae*** | 0.69 | 0.76 |  | 0.71 | 1.13 | 0.072 |  |  | 0.64 | 0.65 |  | 0.80 | 1.06 | 0.602 |  |
| ***Anaerobutyricum hallii*** | 0.90 | 1.60 |  | 0.62 | 1.28 | 0.112 |  |  | 1.22 | 1.98 |  | 0.67 | 1.25 | 0.250 |  |
| ***Bacteroides xylanisolvens*** | 0.75 | 1.71 |  | 0.64 | 1.34 | 0.536 |  |  | 0.17 | 0.23 |  | 0.76 | 1.58 | 0.992 |  |
| ***Bacteroides thetaiotaomicron*** | 0.46 | 0.73 |  | 0.72 | 1.35 | 0.536 |  |  | 0.28 | 0.36 |  | 0.68 | 1.02 | 0.139 |  |
| ***Roseburia intestinalis*** | 0.56 | 1.29 |  | 0.60 | 1.33 | 0.455 |  |  | 0.54 | 1.28 |  | 0.48 | 1.36 | 0.643 |  |
| ***Parasutterella excrementihominis*** | 0.34 | 0.50 |  | 0.56 | 0.82 | 0.861 |  |  | 0.51 | 0.63 |  | 0.54 | 0.83 | 0.204 |  |
| ***Bacteroides caccae*** | **0.46** | **0.91** |  | **0.53** | **1.34** | **0.043** | ***** |  | 0.66 | 1.27 |  | 0.64 | 1.57 | 0.057 |  |
| ***Drancourtella massiliensis*** | 0.46 | 0.76 |  | 0.49 | 0.51 | 0.232 |  |  | **0.34** | **0.89** |  | **0.48** | **0.52** | **0.028** | ***** |
| ***Dorea formicigenerans*** | **0.62** | **0.59** |  | **0.36** | **0.58** | **0.002** | ****** |  | **0.74** | **0.61** |  | **0.44** | **0.64** | **0.018** | ***** |
| ***Megamonas hypermegale*** | **0.59** | **1.71** |  | **0.34** | **2.02** | **0.033** | ***** |  | 0.69 | 1.92 |  | 0.46 | 2.44 | 0.219 |  |
| ***Escherichia coli*** | 0.25 | 0.63 |  | 0.41 | 1.04 | 0.084 |  |  | 0.40 | 0.95 |  | 0.49 | 1.13 | 0.655 |  |
| ***Veillonella ratti*** | 0.23 | 0.54 |  | 0.43 | 0.75 | 0.065 |  |  | 0.13 | 0.36 |  | 0.46 | 0.85 | 0.151 |  |
| ***Bacteroides massiliensis*** | 0.46 | 1.14 |  | 0.31 | 1.54 | 0.153 |  |  | **0.39** | **0.84** |  | **0.23** | **1.40** | **0.030** | ***** |
| ***Alistipes putredinis*** | **0.50** | **0.59** |  | **0.26** | **0.52** | **<0.001** | ******* |  | **0.50** | **0.61** |  | **0.30** | **0.55** | **0.030** | ***** |
| ***Turicibacter sanguinis*** | 0.26 | 0.41 |  | 0.33 | 0.77 | 0.608 |  |  | 0.29 | 0.49 |  | 0.30 | 0.67 | 0.795 |  |
| ***Clostridium saudiense*** | 0.16 | 0.29 |  | 0.30 | 0.99 | 0.593 |  |  | 0.14 | 0.20 |  | 0.16 | 0.46 | 0.319 |  |
| ***Ruminococcus lactaris*** | **0.29** | **0.62** |  | **0.24** | **0.63** | **0.048** | ***** |  | 0.18 | 0.45 |  | 0.19 | 0.54 | 0.318 |  |
| ***Bifidobacterium breve*** | 0.07 | 0.17 |  | 0.29 | 1.02 | 0.164 |  |  | 0.05 | 0.15 |  | 0.39 | 1.20 | 0.111 |  |
| ***Roseburia faecis*** | 0.20 | 0.37 |  | 0.25 | 1.32 | 0.536 |  |  | 0.16 | 0.41 |  | 0.25 | 1.60 | 0.992 |  |
| ***[Eubacterium] eligens*** | 0.20 | 0.28 |  | 0.25 | 0.47 | 0.519 |  |  | 0.20 | 0.24 |  | 0.18 | 0.42 | 0.057 |  |
| ***Eubacterium ventriosum*** | **0.24** | **0.29** |  | **0.23** | **0.40** | **0.048** | ***** |  | **0.22** | **0.23** |  | **0.22** | **0.42** | **0.034** | ***** |
| ***Bifidobacterium catenulatum*** | 0.03 | 0.12 |  | 0.28 | 0.94 | 0.536 |  |  | 0.01 | 0.03 |  | 0.23 | 0.71 | 0.643 |  |
| ***Erysipelatoclostridium ramosum*** | **0.10** | **0.15** |  | **0.24** | **0.33** | **<0.001** | ******* |  | **0.08** | **0.14** |  | **0.27** | **0.33** | **0.002** | ****** |
| ***Dialister invisus*** | 0.27 | 0.41 |  | 0.19 | 0.34 | 0.646 |  |  | 0.31 | 0.45 |  | 0.18 | 0.32 | 0.434 |  |
| ***Parabacteroides distasonis*** | 0.18 | 0.34 |  | 0.20 | 0.48 | 0.604 |  |  | 0.07 | 0.11 |  | 0.22 | 0.46 | 0.526 |  |
| ***Lachnospira pectinoschiza*** | 0.14 | 0.31 |  | 0.22 | 0.60 | 0.864 |  |  | 0.11 | 0.25 |  | 0.17 | 0.51 | 0.886 |  |
| ***Phascolarctobacterium faecium*** | 0.20 | 0.42 |  | 0.18 | 0.47 | 0.072 |  |  | 0.24 | 0.46 |  | 0.18 | 0.43 | 0.159 |  |
| ***Roseburia inulinivorans*** | 0.22 | 0.52 |  | 0.17 | 0.41 | 0.153 |  |  | 0.12 | 0.20 |  | 0.10 | 0.26 | 0.168 |  |
| ***Faecalimonas umbilicata*** | **0.04** | **0.17** |  | **0.22** | **0.71** | **0.033** | ***** |  | 0.28 | 1.33 |  | 0.19 | 0.45 | 0.226 |  |
| ***Odoribacter splanchnicus*** | **0.21** | **0.22** |  | **0.16** | **0.30** | **0.011** | ***** |  | **0.19** | **0.20** |  | **0.15** | **0.32** | **0.030** | ***** |
| ***Bifidobacterium bifidum*** | 0.24 | 0.63 |  | 0.14 | 0.45 | 0.274 |  |  | **0.42** | **0.89** |  | **0.11** | **0.44** | **0.033** | ***** |
| ***Prevotella stercorea*** | 0.38 | 2.41 |  | 0.09 | 0.84 | 0.242 |  |  | 0.00 | 0.00 |  | 0.05 | 0.52 | 0.541 |  |
| ***Prevotellamassilia timonensis*** | 0.52 | 2.67 |  | 0.04 | 0.56 | 0.334 |  |  | 0.24 | 1.25 |  | 0.07 | 0.71 | 0.280 |  |
| ***Ruminococcus callidus*** | **0.18** | **0.37** |  | **0.14** | **0.50** | **0.049** | ***** |  | 0.19 | 0.41 |  | 0.10 | 0.37 | 0.111 |  |
| ***[Clostridium] bolteae*** | **0.05** | **0.08** |  | **0.18** | **0.36** | **<0.001** | ******* |  | **0.08** | **0.25** |  | **0.20** | **0.42** | **0.002** | ****** |
| ***Veillonella infantium*** | 0.04 | 0.07 |  | 0.18 | 0.61 | 0.155 |  |  | 0.16 | 0.69 |  | 0.19 | 0.67 | 0.430 |  |
| ***[Clostridium] innocuum*** | **0.10** | **0.26** |  | **0.16** | **0.24** | **0.043** | ***** |  | 0.08 | 0.15 |  | 0.17 | 0.26 | 0.111 |  |
| ***Barnesiella intestinihominis*** | **0.17** | **0.34** |  | **0.12** | **0.36** | **0.033** | ***** |  | **0.22** | **0.41** |  | **0.04** | **0.16** | **0.002** | ****** |
| ***Anaerostipes caccae*** | 0.11 | 0.42 |  | 0.14 | 0.34 | 0.132 |  |  | **0.01** | **0.02** |  | **0.17** | **0.33** | **0.003** | ****** |
| ***Butyricicoccus faecihominis*** | 0.15 | 0.15 |  | 0.12 | 0.14 | 0.193 |  |  | 0.13 | 0.16 |  | 0.12 | 0.14 | 0.992 |  |
| ***Holdemanella biformis*** | 0.36 | 2.28 |  | 0.03 | 0.35 | 0.132 |  |  | 0.59 | 3.10 |  | 0.08 | 0.56 | 0.450 |  |
| ***Bacteroides finegoldii*** | 0.12 | 0.40 |  | 0.12 | 0.66 | 0.072 |  |  | 0.06 | 0.19 |  | 0.18 | 0.82 | 0.583 |  |
| ***Klebsiella pneumoniae*** | 0.12 | 0.52 |  | 0.11 | 0.65 | 0.755 |  |  | 0.16 | 0.55 |  | 0.16 | 0.80 | 0.701 |  |
| ***Bacteroides eggerthii*** | 0.10 | 0.63 |  | 0.12 | 0.65 | 0.544 |  |  | 0.02 | 0.07 |  | 0.17 | 0.82 | 0.333 |  |
| ***Flavonifractor plautii*** | **0.07** | **0.06** |  | **0.11** | **0.10** | **0.043** | ***** |  | **0.06** | **0.07** |  | **0.12** | **0.10** | **0.019** | ***** |
| s.d.: standard deviation. The first and second urines were collected before and after two days of soymilk supplementation, respectively.  ^a^Relative abundances were compared between equol producers and non-producers using the Wilcoxon rank sum test with Benjamin-Hochberg adjustment. **p*<0.05, ***p*<0.01, ****p*<0.001.  Orange asterisks indicate a higher abundance of the species in equol producers. Blue asterisks indicate a lower abundance of the species in equol producers. | | | | | | | | | | | | | | | |

| **Supplementary table 6.** Spearman’s correlation coefficients between the equol/daidzein ratio and the relative abundances of 71 bacterial species with a mean relative abundance of >0.1% | | | | | | |  |
| --- | --- | --- | --- | --- | --- | --- | --- |
|  | The first collected urine samples (n=204) | | | The second collected urine samples (n=126) | | | |
|  | Spearman’s ρ | *p*^a^ |  | Spearman’s ρ | *p*^a^ |  | |
| ***Blautia luti*** | -0.047 | 0.607 |  | -0.043 | 0.704 |  | |
| ***Bacteroides vulgatus*** | -0.049 | 0.607 |  | -0.002 | 0.983 |  | |
| ***Bacteroides dorei*** | -0.015 | 0.838 |  | -0.118 | 0.325 |  | |
| ***Bifidobacterium pseudocatenulatum*** | -0.017 | 0.838 |  | -0.205 | 0.060 |  | |
| ***Faecalibacterium prausnitzii*** | 0.144 | 0.136 |  | 0.047 | 0.704 |  | |
| ***Anaerostipes hadrus*** | 0.039 | 0.646 |  | 0.143 | 0.216 |  | |
| ***Bifidobacterium longum*** | -0.104 | 0.311 |  | -0.205 | 0.060 |  | |
| ***Fusicatenibacter saccharivorans*** | -0.049 | 0.607 |  | -0.080 | 0.488 |  | |
| ***Bacteroides stercoris*** | -0.027 | 0.758 |  | -0.101 | 0.384 |  | |
| ***Romboutsia timonensis*** | 0.104 | 0.311 |  | **0.218** | **0.050** | ***** | |
| ***[Eubacterium] rectale*** | 0.114 | 0.264 |  | **0.349** | **0.001** | ******* | |
| ***Bacteroides uniformis*** | 0.074 | 0.431 |  | 0.020 | 0.888 |  | |
| ***Bacteroides ovatus*** | -0.085 | 0.395 |  | -0.078 | 0.497 |  | |
| ***Prevotella copri*** | 0.078 | 0.414 |  | 0.189 | 0.086 |  | |
| ***Bacteroides fragilis*** | -0.166 | 0.074 |  | -0.206 | 0.060 |  | |
| ***[Ruminococcus] gnavus*** | **-0.226** | **0.012** | ***** | **-0.400** | **0.000** | ******* | |
| ***Bacteroides coprocola*** | 0.059 | 0.545 |  | 0.194 | 0.079 |  | |
| ***Bifidobacterium faecale*** | 0.134 | 0.161 |  | **0.230** | **0.049** | ***** | |
| ***Bifidobacterium adolescentis*** | 0.069 | 0.462 |  | 0.085 | 0.477 |  | |
| ***Streptococcus salivarius*** | 0.078 | 0.414 |  | 0.002 | 0.983 |  | |
| ***Intestinibacter bartlettii*** | -0.044 | 0.621 |  | 0.056 | 0.660 |  | |
| ***Bacteroides plebeius*** | 0.172 | 0.065 |  | **0.237** | **0.045** | ***** | |
| ***Streptococcus equinus*** | -0.101 | 0.318 |  | -0.049 | 0.704 |  | |
| ***[Ruminococcus] torques*** | 0.078 | 0.414 |  | 0.154 | 0.171 |  | |
| ***Streptococcus pasteurianus*** | -0.175 | 0.065 |  | -0.013 | 0.924 |  | |
| ***Collinsella aerofaciens*** | 0.100 | 0.318 |  | 0.130 | 0.267 |  | |
| ***Parabacteroides merdae*** | 0.139 | 0.149 |  | 0.064 | 0.600 |  | |
| ***Anaerobutyricum hallii*** | 0.121 | 0.223 |  | **0.349** | **0.001** | ******* | |
| ***Bacteroides xylanisolvens*** | 0.077 | 0.414 |  | -0.114 | 0.341 |  | |
| ***Bacteroides thetaiotaomicron*** | -0.048 | 0.607 |  | -0.110 | 0.359 |  | |
| ***Roseburia intestinalis*** | 0.096 | 0.318 |  | 0.099 | 0.389 |  | |
| ***Parasutterella excrementihominis*** | -0.016 | 0.838 |  | -0.021 | 0.888 |  | |
| ***Bacteroides caccae*** | **0.201** | **0.032** | * | 0.176 | 0.105 |  | |
| ***Drancourtella massiliensis*** | -0.108 | 0.308 |  | -0.177 | 0.105 |  | |
| ***Dorea formicigenerans*** | **0.242** | **0.009** | ****** | **0.324** | **0.002** | ****** | |
| ***Megamonas hypermegale*** | 0.157 | 0.092 |  | **0.237** | **0.045** | ***** | |
| ***Escherichia coli*** | -0.098 | 0.318 |  | -0.142 | 0.217 |  | |
| ***Veillonella ratti*** | -0.099 | 0.318 |  | -0.101 | 0.384 |  | |
| ***Bacteroides massiliensis*** | 0.105 | 0.311 |  | 0.124 | 0.293 |  | |
| ***Alistipes putredinis*** | **0.352** | **0.000** | ******* | **0.223** | **0.050** | * | |
| ***Turicibacter sanguinis*** | 0.006 | 0.934 |  | -0.082 | 0.488 |  | |
| ***Clostridium saudiense*** | 0.041 | 0.630 |  | 0.213 | 0.056 |  | |
| ***Ruminococcus lactaris*** | 0.156 | 0.092 |  | 0.172 | 0.112 |  | |
| ***Bifidobacterium breve*** | -0.163 | 0.077 |  | **-0.232** | **0.049** | ***** | |
| ***Roseburia faecis*** | 0.080 | 0.414 |  | 0.011 | 0.928 |  | |
| ***[Eubacterium] eligens*** | 0.041 | 0.630 |  | 0.208 | 0.060 |  | |
| ***Eubacterium ventriosum*** | **0.230** | **0.012** | ***** | **0.361** | **0.001** | ******* | |
| ***Bifidobacterium catenulatum*** | -0.077 | 0.414 |  | -0.101 | 0.384 |  | |
| ***Erysipelatoclostridium ramosum*** | **-0.268** | **0.002** | ****** | **-0.307** | **0.004** | ****** | |
| ***Dialister invisus*** | 0.051 | 0.607 |  | 0.087 | 0.470 |  | |
| ***Parabacteroides distasonis*** | -0.023 | 0.793 |  | -0.110 | 0.359 |  | |
| ***Lachnospira pectinoschiza*** | 0.031 | 0.719 |  | 0.084 | 0.477 |  | |
| ***Phascolarctobacterium faecium*** | 0.139 | 0.149 |  | 0.102 | 0.384 |  | |
| ***Roseburia inulinivorans*** | 0.125 | 0.205 |  | **0.221** | **0.050** | ***** | |
| ***Faecalimonas umbilicata*** | -0.184 | 0.059 |  | -0.182 | 0.102 |  | |
| ***Odoribacter splanchnicus*** | **0.203** | **0.031** | ***** | **0.224** | **0.050** | ***** | |
| ***Bifidobacterium bifidum*** | 0.068 | 0.462 |  | **0.219** | **0.050** | ***** | |
| ***Prevotella stercorea*** | 0.069 | 0.462 |  | 0.043 | 0.704 |  | |
| ***Prevotellamassilia timonensis*** | 0.054 | 0.586 |  | -0.015 | 0.916 |  | |
| ***Ruminococcus callidus*** | 0.174 | 0.065 |  | 0.177 | 0.105 |  | |
| ***[Clostridium] bolteae*** | **-0.310** | **0.000** | ******* | **-0.384** | **0.000** | ******* | |
| ***Veillonella infantium*** | -0.046 | 0.607 |  | -0.181 | 0.102 |  | |
| ***[Clostridium] innocuum*** | **-0.229** | **0.012** | ***** | **-0.228** | **0.049** | ***** | |
| ***Barnesiella intestinihominis*** | 0.168 | 0.072 |  | **0.244** | **0.045** | ***** | |
| ***Anaerostipes caccae*** | -0.178 | 0.065 |  | **-0.377** | **0.000** | ******* | |
| ***Butyricicoccus faecihominis*** | 0.136 | 0.154 |  | -0.047 | 0.704 |  | |
| ***Holdemanella biformis*** | 0.058 | 0.550 |  | 0.055 | 0.660 |  | |
| ***Bacteroides finegoldii*** | 0.093 | 0.330 |  | 0.203 | 0.062 |  | |
| ***Klebsiella pneumoniae*** | 0.094 | 0.330 |  | -0.133 | 0.255 |  | |
| ***Bacteroides eggerthii*** | 0.097 | 0.318 |  | 0.045 | 0.704 |  | |
| ***Flavonifractor plautii*** | -0.173 | 0.065 |  | **-0.238** | **0.045** | * | |
| The first and second urines were collected before and after two days of soymilk supplementation, respectively.  ^a^Benjamin-Hochberg adjusted *p* values. *p<0.05, **p<0.01, ***p<0.001.  Orange asterisks indicate a positive association between the relative abundances of species and the equol/daidzein ratio. Blue asterisks indicate an inverse association between the relative abundances of species and the equol/daidzein ratio. | | | | | | |  |

| **Supplementary Table 7.** Relative abundances of 12 potentially equol-producing species according to equol production in the first urine samples. | | | | | | | | | |  |
| --- | --- | --- | --- | --- | --- | --- | --- | --- | --- | --- |
|  | Detection rate (%) | Equol (+), *n*=53 | |  | Equol (-), *n*=169 | |  |  | |  |
| Species |  | mean | s.d. |  | mean | s.d. |  | *p*^a^ | |  |
| ***Bifidobacterium longum*** | 97.3% | 2.337 | 2.019 |  | 3.314 | 3.341 |  | 0.173 | |  |
| ***Bacteroides ovatus*** | 77.6% | 1.008 | 1.869 |  | 1.877 | 2.672 |  | 0.103 | |  |
| ***Bifidobacterium breve*** | 52.9% | 0.065 | 0.169 |  | 0.290 | 1.020 |  | 0.078 | |  |
| ***Lacticaseibacillus paracasei*** | 19.7% | 0.066 | 0.186 |  | 0.067 | 0.284 |  | 0.449 | |  |
| ***Limosilactobacillus mucosae*** | 4.0% | 0.031 | 0.142 |  | 0.032 | 0.286 |  | 0.141 | |  |
| ***Asaccharobacter celatus*** | 26.0% | **0.049** | **0.065** |  | **0.003** | **0.012** |  | **<0.001** | |  |
| ***Latilactobacillus sakei*** | 1.4% | 0.000 | 0.000 |  | 0.012 | 0.160 |  | 0.333 | |  |
| ***Slackia isoflavoniconvertens*** | 2.2% | **0.009** | **0.044** |  | **0.001** | **0.010** |  | **0.003** | |  |
| ***Streptococcus intermedius*** | 16.6% | 0.002 | 0.005 |  | 0.002 | 0.005 |  | 0.503 | |  |
| ***Finegoldia magna*** | 8.1% | 0.000 | 0.002 |  | 0.002 | 0.012 |  | 0.176 | |  |
| ***Lactococcus garvieae*** | 1.4% | 0.000 | 0.001 |  | 0.002 | 0.025 |  | 0.709 | |  |
| ***Proteus mirabilis*** | 2.2% | 0.000 | 0.001 |  | 0.001 | 0.005 |  | 0.834 | |  |
| s.d.: standard deviation. Equol production was determined in urine collected before soymilk supplementation.  ^a^Relative abundances were compared between equol producers and non-producers using the Wilcoxon rank sum test. | | | | | | | | |  | |

**Supplementary Figure 1.** **Flowchart of the study participants**

A public elementary and a junior high school students in Gifu, Japan, 2013-2016 **(n=1563)**

7–8 years (the 2nd grade) 10–11 years (the 5th grade) 13–14 years (the 8th grade)

(n=427) (n=477) (n=659)

Participants **(n=1332, 85,2%)**

7–8 years 10–11 years 13–14 years

Total **n= 377 (88.3%) n=402 (84.3%) n=553 (83.9%)**

2013 n=104 n=108 n=146

2014 n=97 n=105 n=145

2015 n=90 n=92 n=133

2016 n=86 n=97 n=129

The 2nd and 5th grade students in the 2013 survey **(n=212)**

FFQ was unmeasured **(n=4)**

Urines were uncollected **(n=6)**

**Excluded**

Participants included in the analysis **(n=1110)**

7–8 years (n=268) 10–11 years (n=292) 13–14 years (n=550)

Urine collected before and after soymilk supplementation

1106 and 972 samples at the first (before) and second (after) collection

**→** 6 samples unable to determine equol production

**1100 and 972 samples** at the first and second collection with determination of equol production

*→ For analysis on isoflavone exposure and equol production*

Feces collected in 2016 **(n=231)**

**→ (Excluded)** Those who took antibiotics within 3 days before collection **(n=8)**

**223 feces**

**222** with equol production in the first urine samples

**133** with equol production in the second urine samples

*→ For analysis on gut microbiome and equol production*

**Supplementary Figure 2.** **Indices of gut microbiota α-diversity according to** **sex and age group**

S2-a. Observed OTU number S2-d. Observed OTU number

300

250

200

150

100

300

250

200

150

100

**
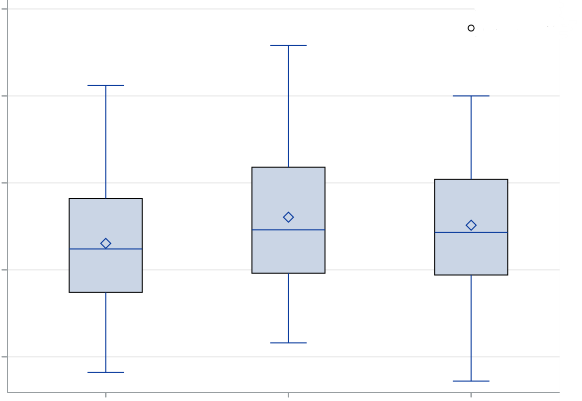

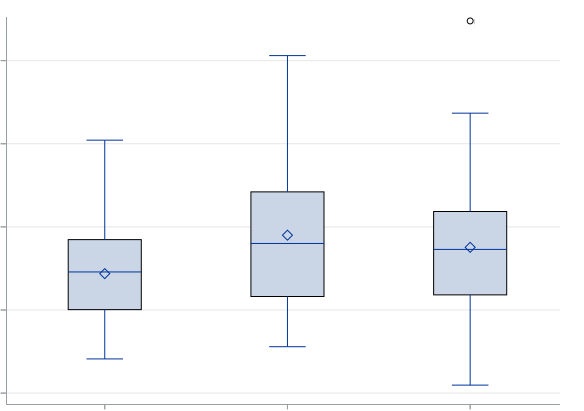

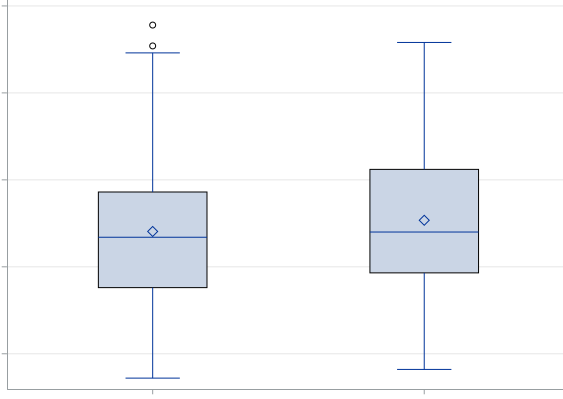
**

*p*=0.078

*p*=0.26

500

400

300

200

100

7-8 y. 10-11 y. 13-14 y.

male female

S2-b. Chao1 S2-e. Chao1

1-d. Chao1

1-d. Chao1

1-d. Chao1

1-d. Chao1

1-d. Chao1

1-d. Chao1

**
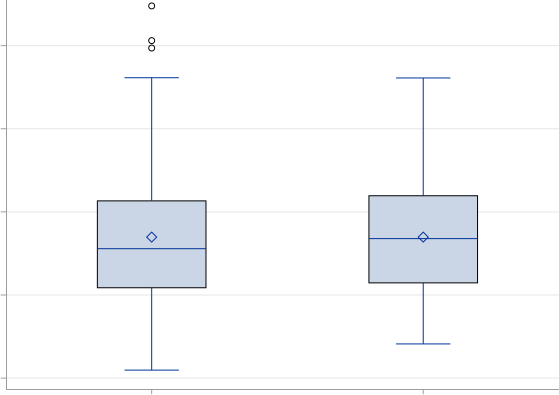
**

*p*=0.001

*p*=1.00

500

400

300

200

100

7-8 y. 10-11 y. 13-14 y.

male female

S2-c. Shannon S2-f. Shannon

**
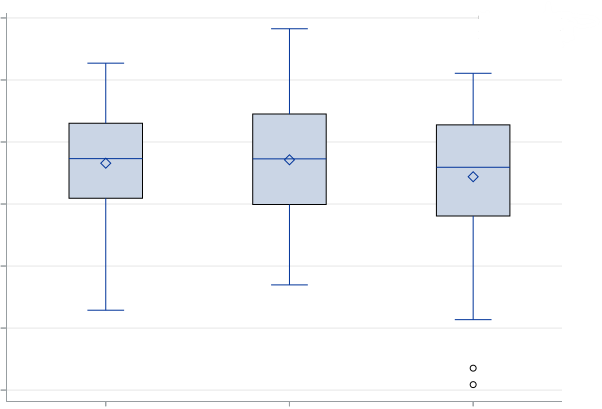

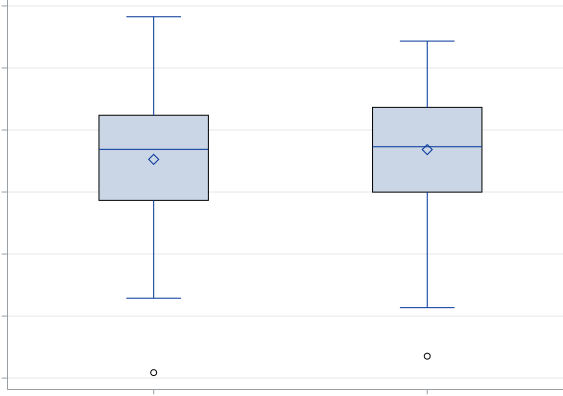
**

*p*=0.21

*p*=0.23

6.0

5.5

5.0

4.5

4.0

3.5

3.0

6.0

5.5

5.0

4.5

4.0

3.5

3.0

7-8 y. 10-11 y. 13-14 y.

male female

S2-a, S2-b, S2-c. Comparison of gut microbiome α-diversity between male (n=107) and female (n=116); S2-d, S2-e, S2-f. Comparison of gut microbiome α-diversity between 7-8 years old (n=76), 10-11 years old (n=77), and 13-14 years old (n=70).

*P* values for the comparison were assessed using *t*-test (for sex) or analysis of variance (for age group).

**Supplementary Figure 3.** **Indices of gut microbiota α-diversity according to tertiles of isoflavone intake**

S3-a. Observed OTU number

300

250

200

150

100

**
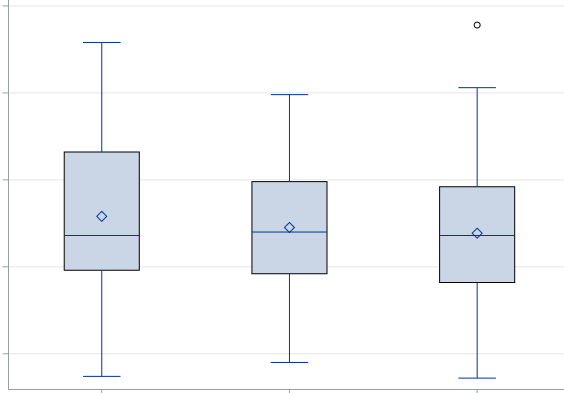
**

*p*=0.46

Low middle high

S3-b. Chao1

1-d. Chao1

1-d. Chao1

1-d. Chao1

1-d. Chao1

1-d. Chao1

1-d. Chao1

**
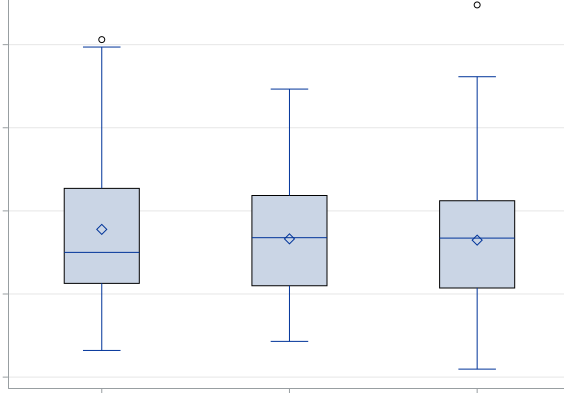
**

*p*=0.84

500

400

300

200

100

Low middle high

S3-c. Shannon

**
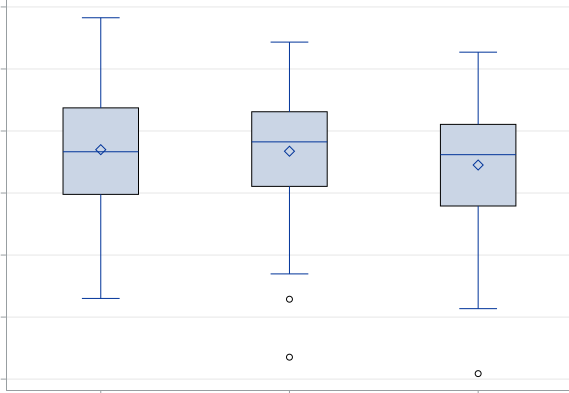
**

*p*=0.18

6.0

5.5

5.0

4.5

4.0

3.5

3.0

Low middle high

S3-a, S3-b, S3-c. Comparison of gut microbiome α-diversity between those with isoflavone intake of low (<26 mg/d), middle (26-34 mg/d), and high (>34 mg/d).

Isoflavone intake was adjusted for total energy by using the residual method proposed by Willett.

*P* values for the comparison between tertiles of isoflavone intake were assessed by analysis of covariance with adjustment for survey time, sex, and age.

**Supplementary Figure 4.** **Comparison of gut microbiota α-diversity between equol producers (n=53) and non-producers (n=169) in the first collected urine samples by analysis of covariance with adjustment for survey time, sex, and age**

S4-a. Observed OTU number

300

250

200

150

100

**
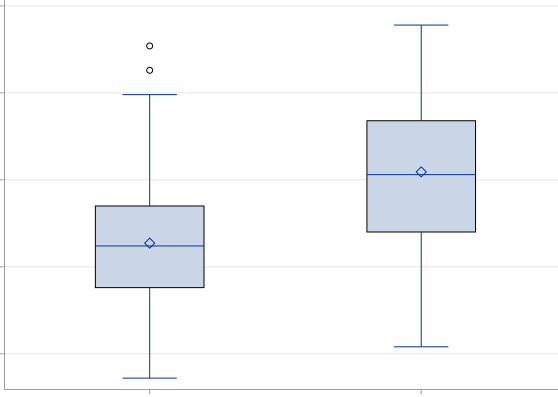
**

*p*<0.001

Equol(-) Equol(+)

S4-b. Chao1

1-d. Chao1

1-d. Chao1

1-d. Chao1

1-d. Chao1

1-d. Chao1

1-d. Chao1

**
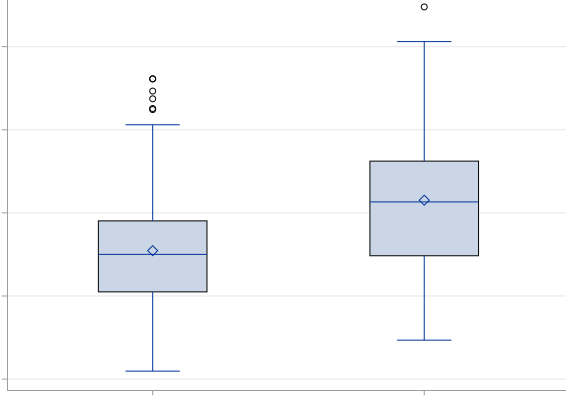
**

*p*<0.001

500

400

300

200

100

Equol(-) Equol(+)

S4-c. Shannon

**
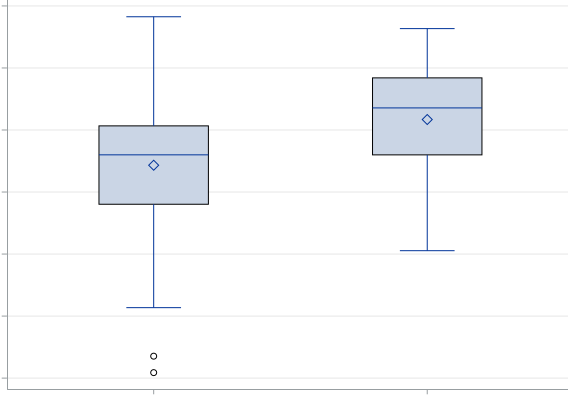
**

*p* <0.001

6.0

5.5

5.0

4.5

4.0

3.5

3.0

Equol(-) Equol(+)

Equol production was determined in urine collected before soymilk supplementation.

**Supplementary Figure 5.** **Comparison of Chao1 between equol producers and non-producers by age group**

400

300

200

S5-a. 7-8 years old

**
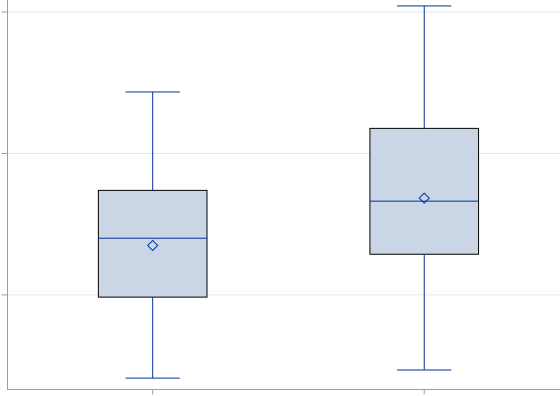
**

*p*=0.041

Equol(-) Equol(+)

S5-b. 10-11 years old S5-d. 10-11 years old

1-d. Chao1

1-d. Chao1

1-d. Chao1

1-d. Chao1

1-d. Chao1

1-d. Chao1

500

400

300

200

500

400

300

200

**
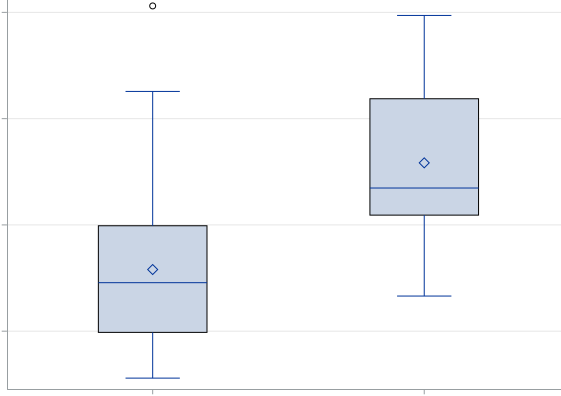

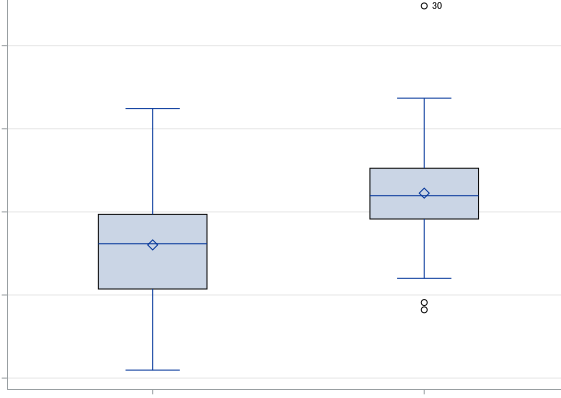

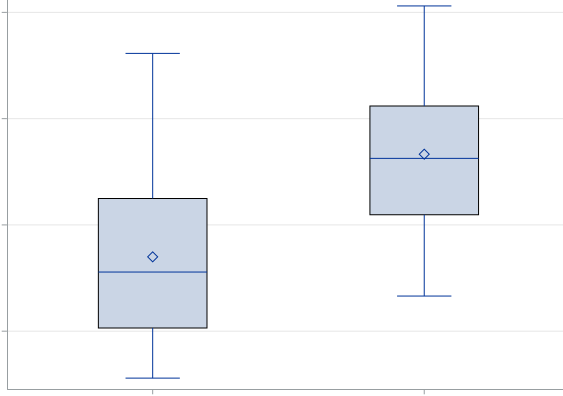
**

*p*<0.001

*p*<0.001

500

400

300

200

100

Equol(-) Equol(+)

Equol(-) Equol(+)

S5-c. 13-14 years old S5-e. 13-14 years old

**
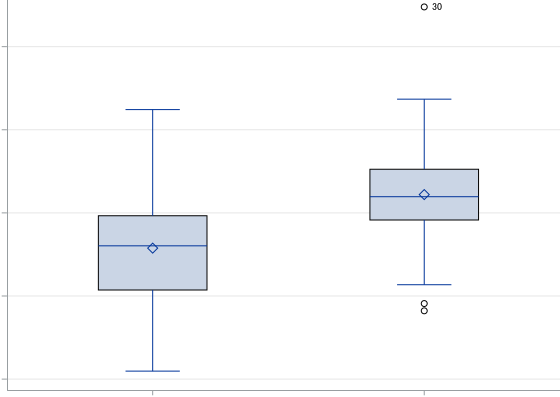
**

*p*=0.002

*p*=0.003

500

400

300

200

100

Equol(-) Equol(+)

Equol(-) Equol(+)

S5-a, S5-b, S5-c. Chao1 of equol producers and non-producers in the first urines among 7-8, 10-11, and 13-14 years old; S5-d, S5-e. Chao1 of equol producers and non-producers in the second urines among 10-11 and 13-14 years old.

The first and second urines were collected before and after two days of soymilk supplementation, respectively. *P* values for the comparison between equol producer and non-producer were assessed by analysis of covariance after adjustments for survey time, sex, and age.

**Supplementary Figure 6. Principal coordinate analysis (PCoA) plots based on UniFrac distances of the gut microbiota for the comparison between equol production (n=53) and non-production (n=169) in the first urines**


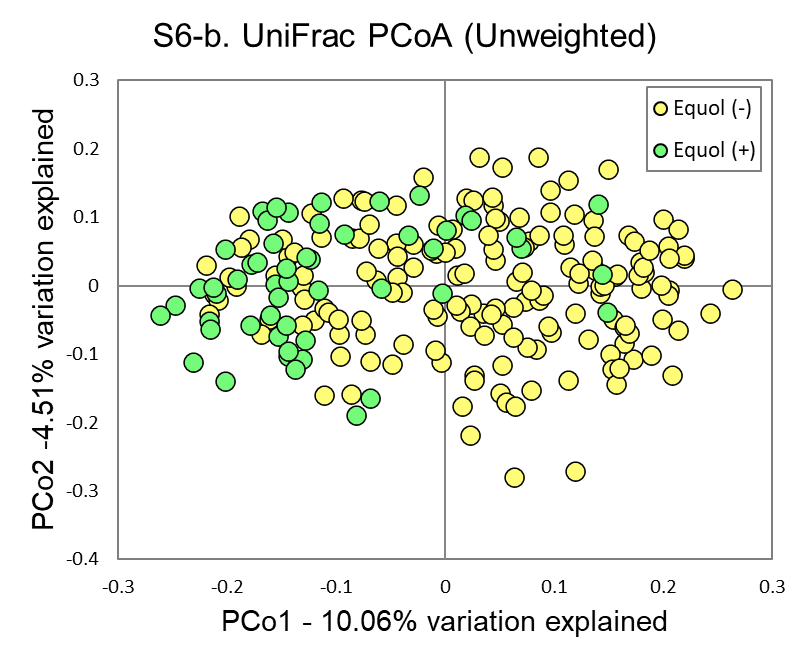

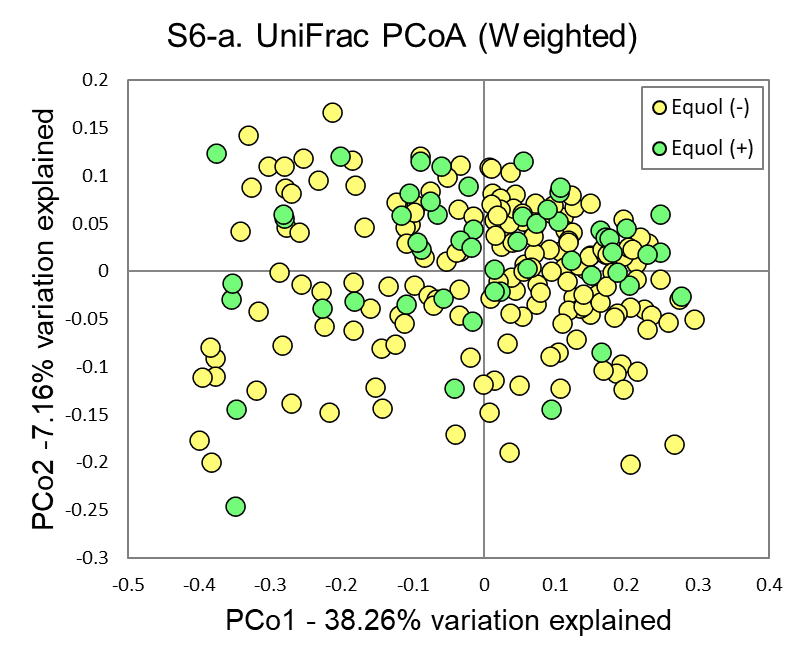


R^2^: 0.0276, *p*<0.001 by PERMANOVA

R^2^: 0.0124, *p*=0.021 by PERMANOVA

Equol production was determined in urine collected before soymilk supplementation.

**Supplementary Figure 7.** **Principal coordinate analysis (PCoA) plots based on UniFrac distances of the gut microbiota for the comparison between equol production and non-production by sex**


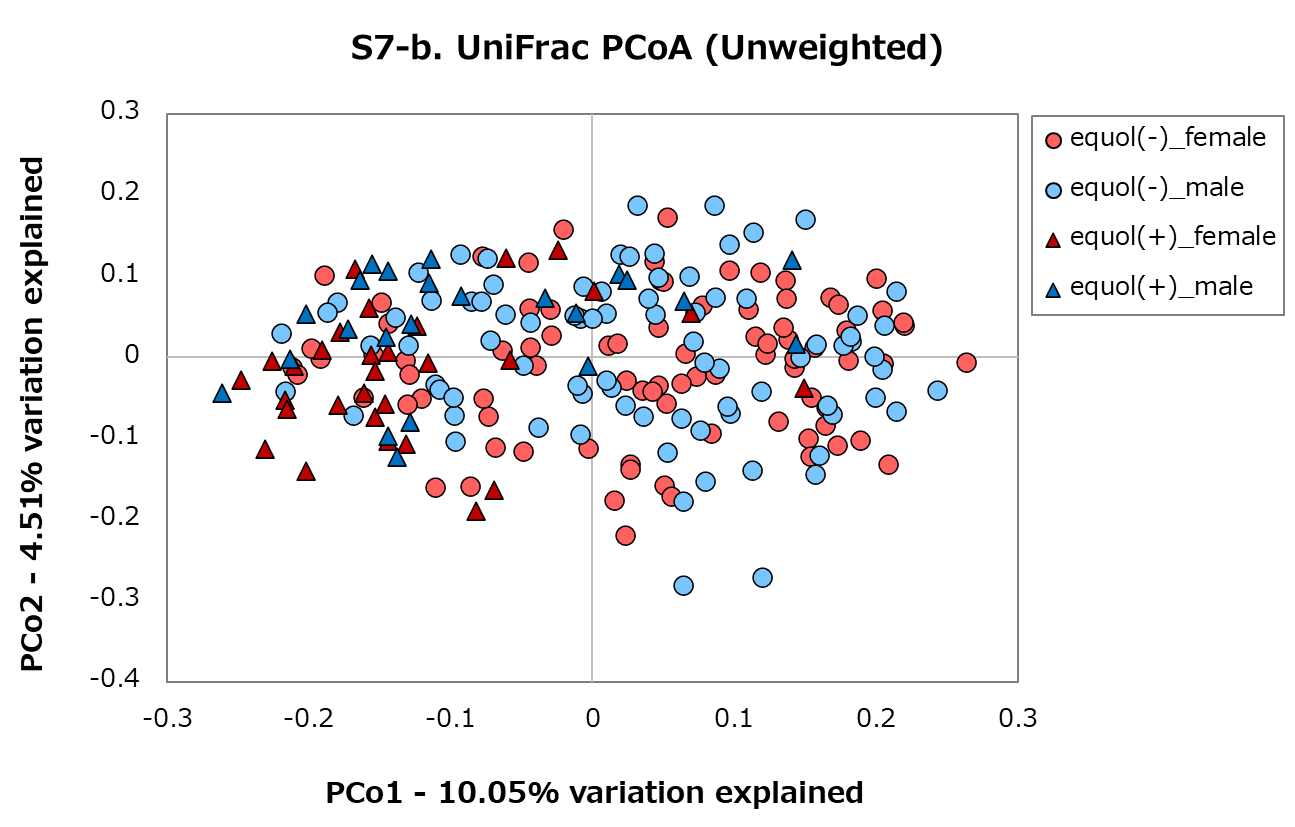

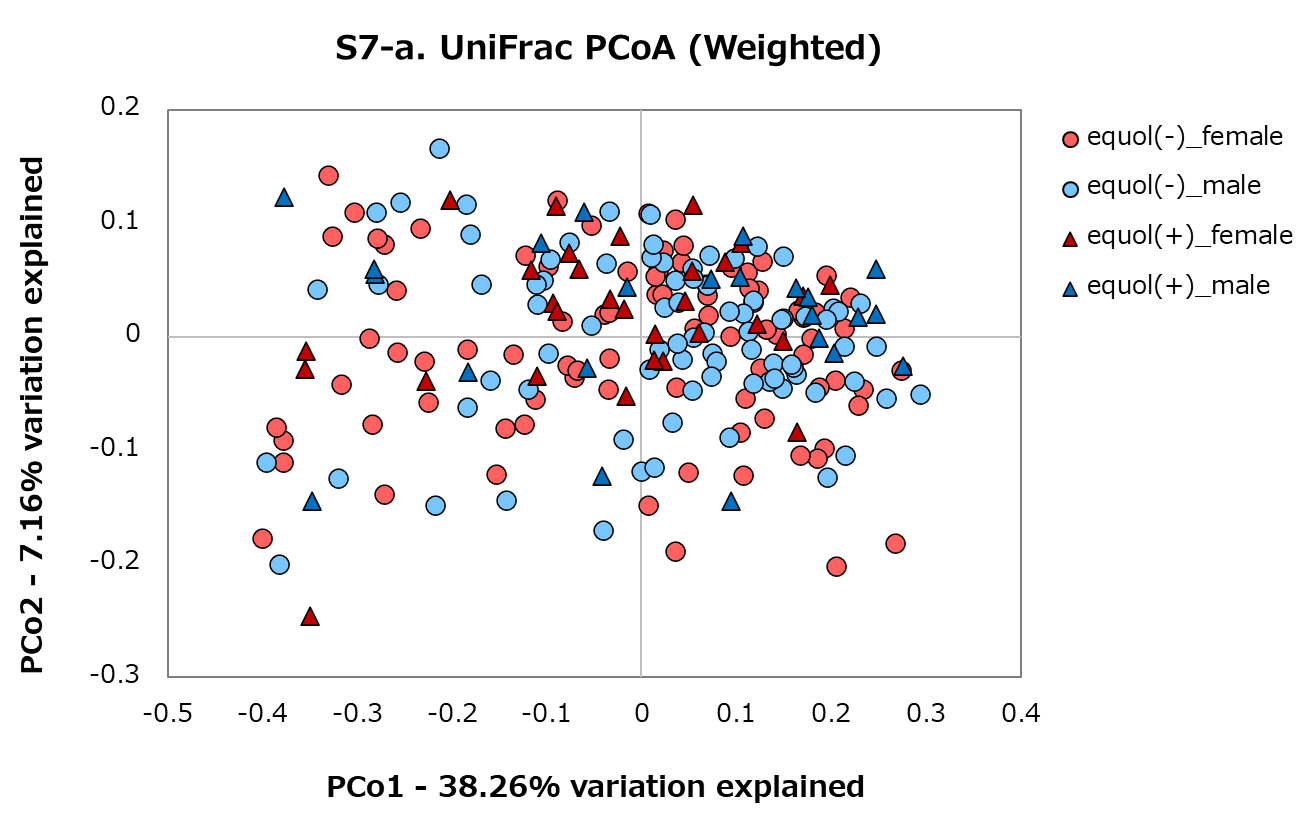


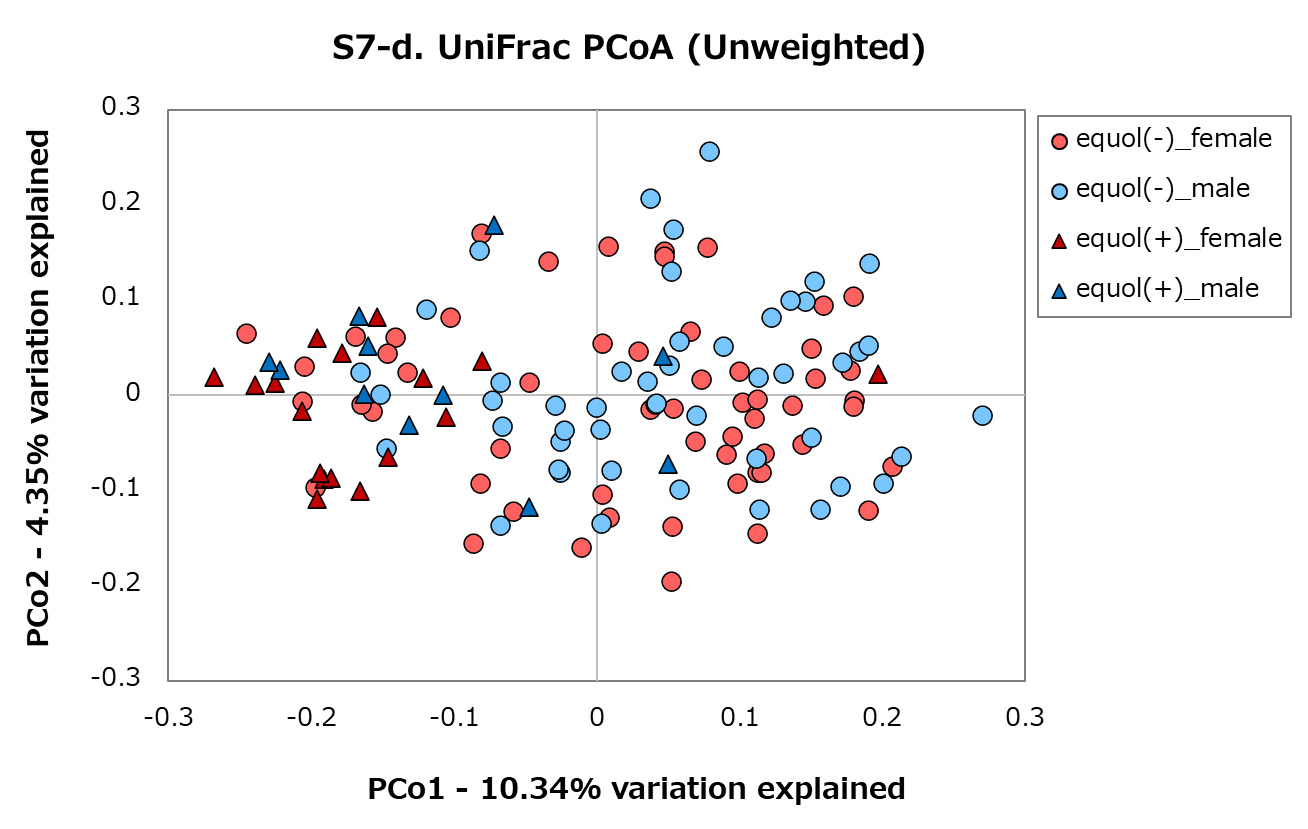

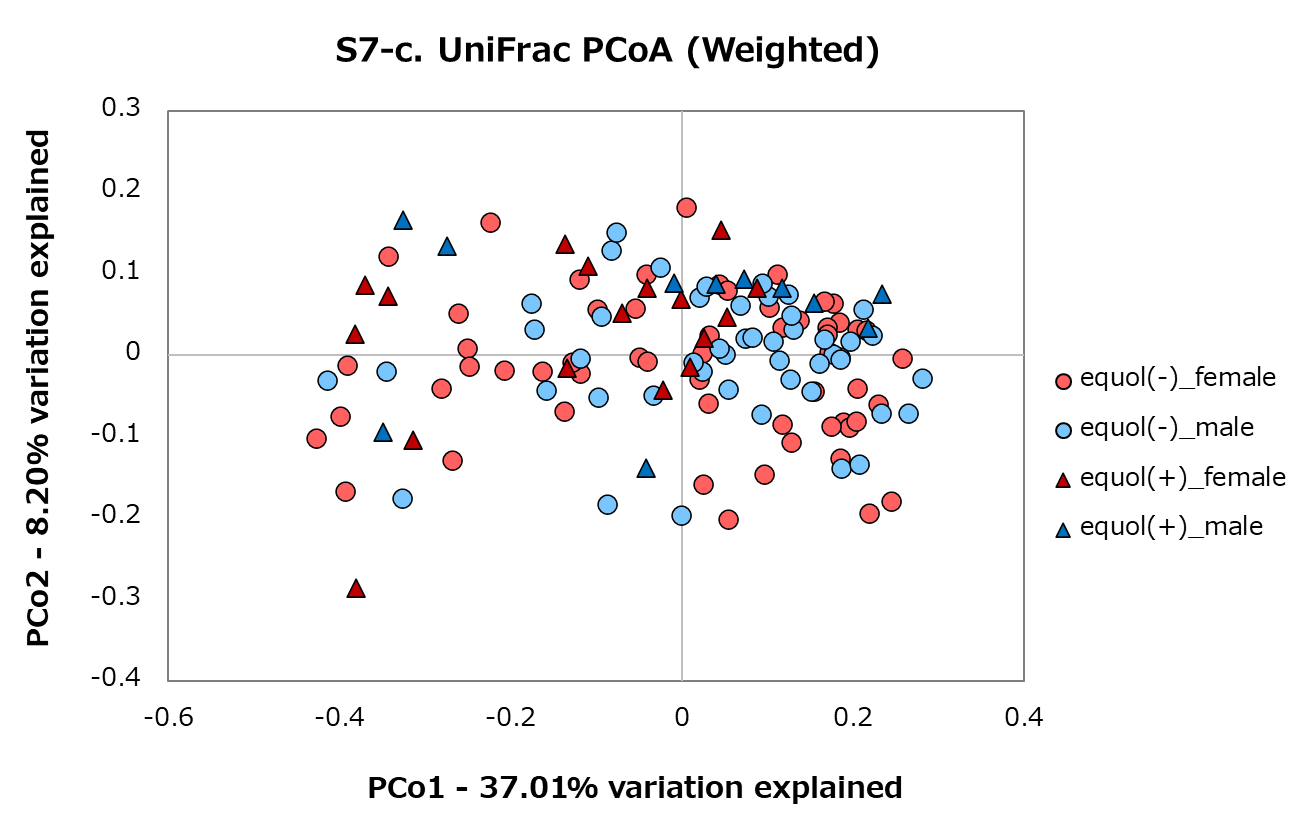


| **Permanova results of β-diversity according to equol production status by sex** | | | | | | |
| --- | --- | --- | --- | --- | --- | --- |
|  |  | Weighted UniFrac | |  | Unweighted UniFrac | |
| Equol, (+) vs. (-) | *n* | R^2^ | *p* |  | R^2^ | *p* |
| the first urines (S7-a, S7-b) |  |  |  |  |  |  |
| male | 23 vs. 84 | 0.015 | 0.147 |  | 0.023 | <0.001 |
| female | 30 vs. 85 | 0.018 | 0.073 |  | 0.040 | <0.001 |
| the second urines (S7-c, S7-d) |  |  |  |  |  |  |
| male | 11 vs. 48 | 0.043 | 0.032 |  | 0.037 | <0.001 |
| female | 17 vs. 57 | 0.049 | 0.006 |  | 0.047 | <0.001 |

S7-a, S7-b. Equol production (+) vs. (-) in the first urines; S7-c, S7-d. Equol production (+) vs. (-) in the second urines.

The first and second urines were collected before and after two days of soymilk supplementation, respectively.

**Supplementary Figure 8.** **Principal coordinate analysis (PCoA) plots based on UniFrac distances of the gut microbiota for the comparison between equol producers and non-producers by age group**


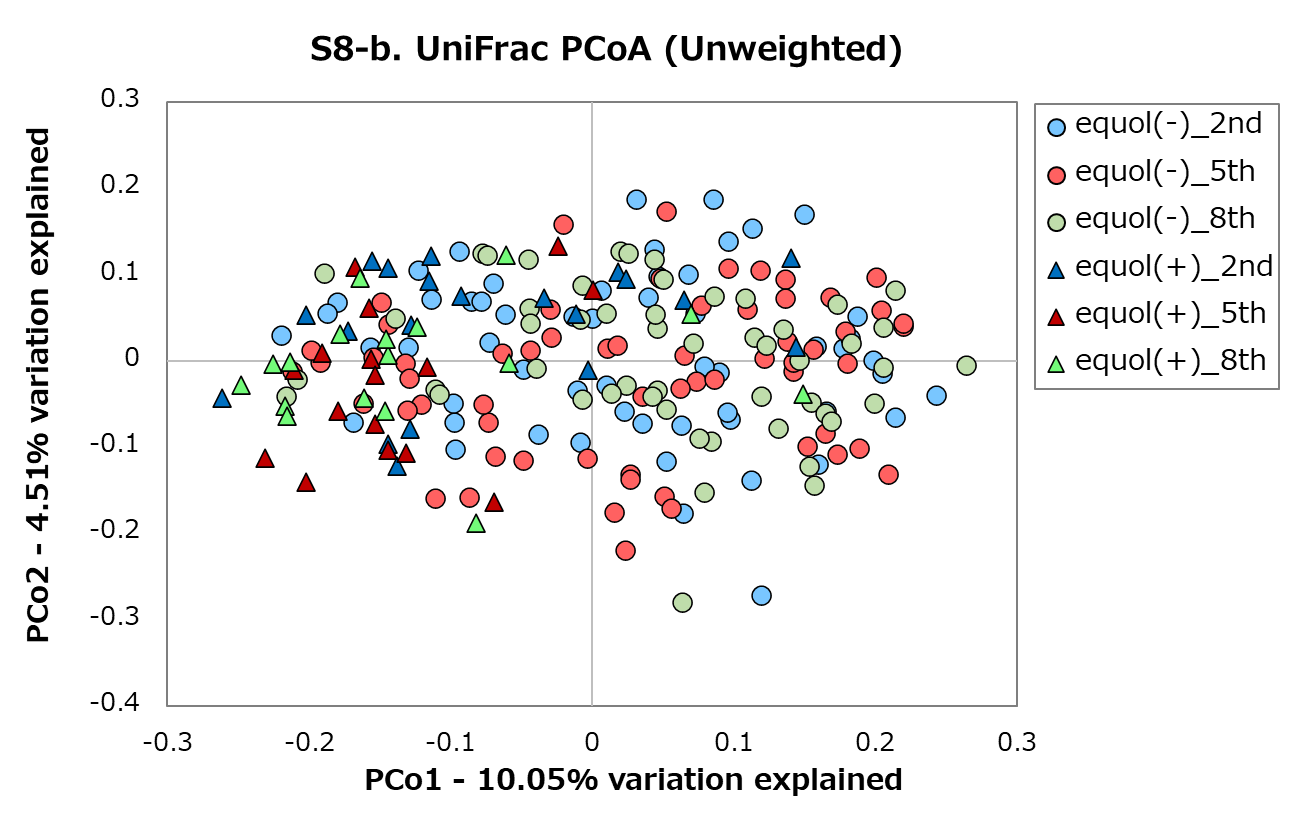

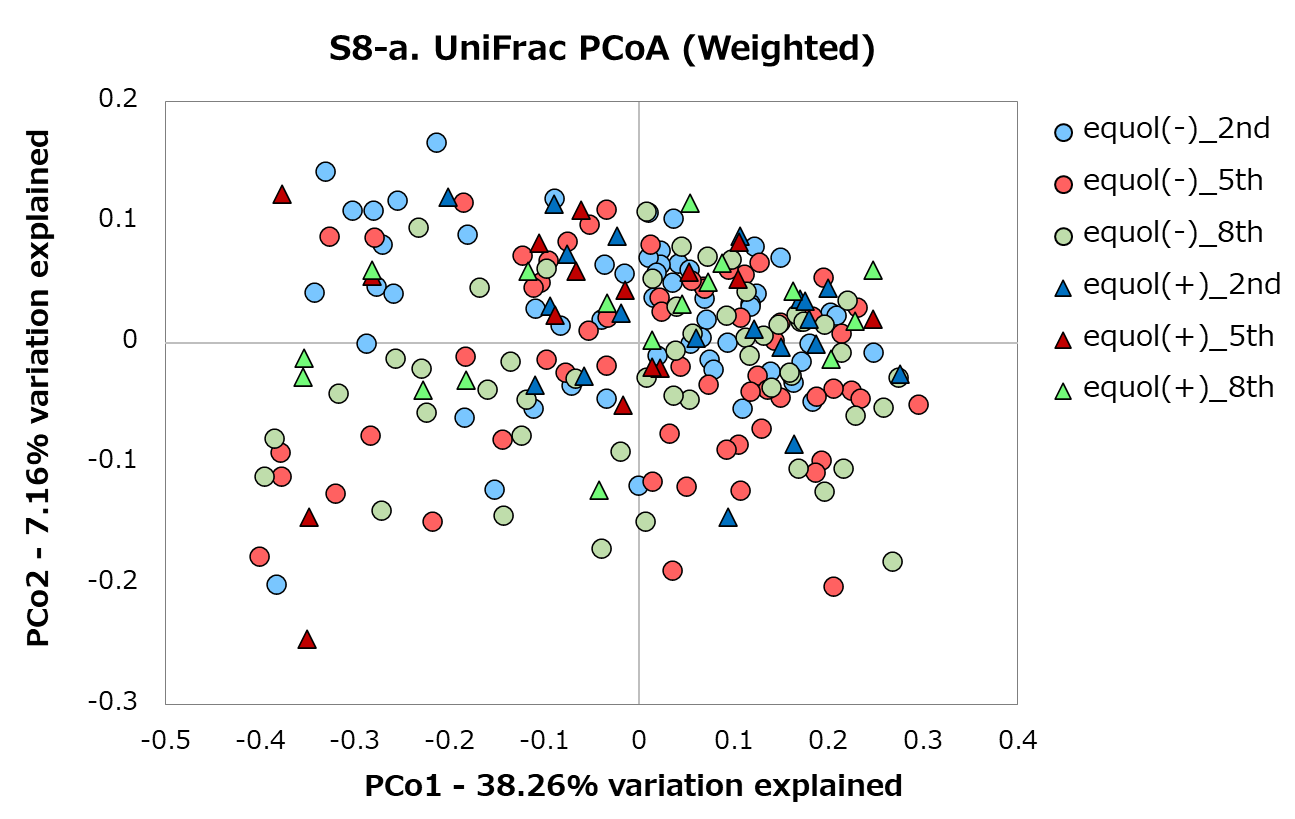


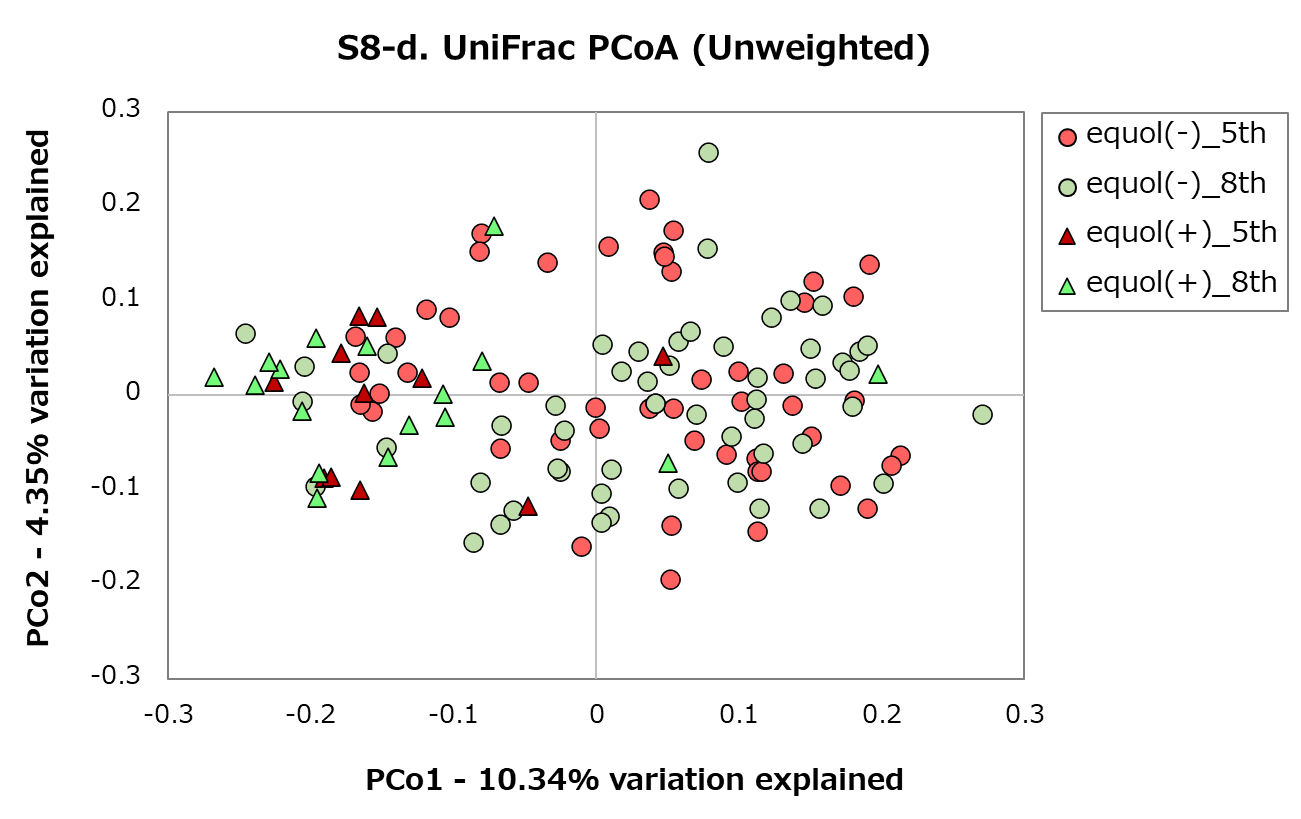

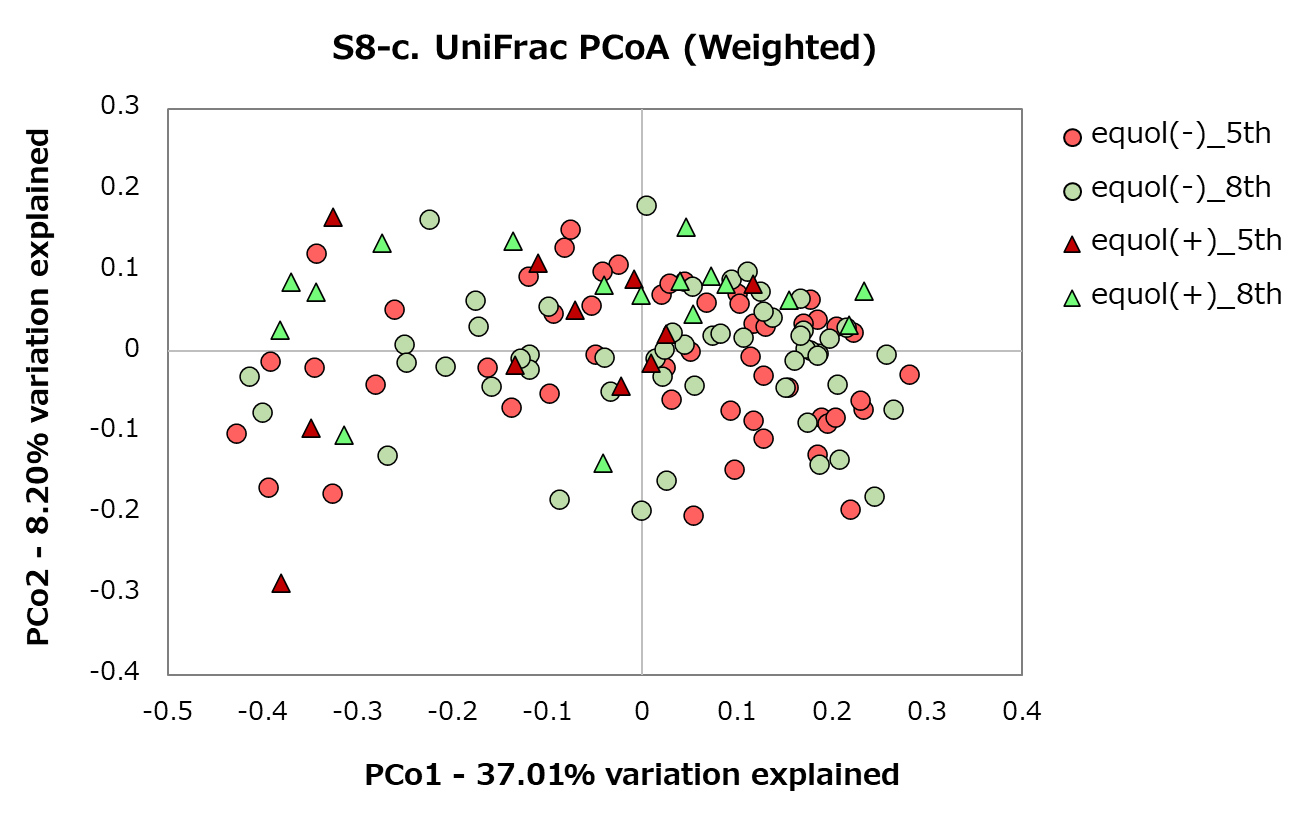


e se

| **Permanova results of β-diversity according to equol production status by age group** | | | | | | |
| --- | --- | --- | --- | --- | --- | --- |
|  | | | | | | |
|  |  | Weighted UniFrac | |  | Unweighted UniFrac | |
| Equol, (+) vs. (-) | *n* | R^2^ | *p* |  | R^2^ | *p* |
| the first urines (S8-a, S8-b) |  |  |  |  |  |  |
| 7-8 years (2^nd^) | 20 vs. 56 | 0.036 | 0.030 |  | 0.027 | <0.001 |
| 10-11 years (5^th^) | 16 vs. 61 | 0.037 | 0.026 |  | 0.040 | <0.001 |
| 13-14 years (8^th^) | 17 vs. 52 | 0.029 | 0.085 |  | 0.047 | <0.001 |
| the second urines (S8-c, s S8-d) |  |  |  |  |  |  |
| 10-11 years (5^th^) | 11 vs. 52 | 0.046 | 0.024 |  | 0.040 | <0.001 |
| 13-14 years (8^th^) | 17 vs. 53 | 0.043 | 0.015 |  | 0.047 | <0.001 |

S8-a, S8-b. Equol production (+) vs. (-) in the first urines; S8-c, S8-d. Equol production (+) vs. (-) in the second urines.

The first and second urines were collected before and after two days of soymilk supplementation, respectively.

**Supplementary Figure 9.** **Principal coordinate analysis (PCoA) plots based on UniFrac distances of the gut microbiota according to isoflavone intake (*n*=223)**

**S9-a. UniFrac PCoA (Weighted) S9-b. UniFrac PCoA (Unweighted)**

R^2^: 0.006, *p*=0.055 by PERMANOVA

R^2^: 0.003, *p*=0.67 by PERMANOVA

S9-a, S9-b. Principal coordinate analysis (PCoA) plots based on UniFrac distances of the gut microbiomes with isoflavone intake.

Isoflavone intake was adjusted for total energy by using the residual method proposed by Willett.
